# Bidirectional Relations Between Spatial and Mathematics Skills in Elementary School Children: The Role of Domain-Specific Anxieties

**DOI:** 10.3390/jintelligence14050087

**Published:** 2026-05-19

**Authors:** Elyssa A. Geer, Connie Barroso, Colleen M. Ganley

**Affiliations:** 1 Anita Zucker Center for Excellence in Early Childhood Studies, University of Florida, Gainesville, FL 32611, USA; 2 Department of Educational Psychology, Texas A&M University, College Station, TX 77843, USA; cbarroso@tamu.edu; 3 Department of Psychology and Learning Systems Institute, Florida State University, Tallahassee, FL 32306, USA; ganley@psy.fsu.edu

**Keywords:** spatial cognition, mental rotation, mathematics, mathematics anxiety, spatial anxiety, domain specificity

## Abstract

Research has demonstrated a link between spatial and mathematics skills, although less research has addressed the directionality of this relation and what factors may impact the spatial–mathematics link. Two such factors may be spatial and mathematics anxiety, which are related to performance in their respective domains, but may also play a cross-domain role in the relation between spatial and mathematics skills. Importantly, these relations are prudent to investigate in children when mathematics and spatial skills are early in their development. To bridge the gap, the present study examined the direction of the relation between spatial and mathematics skills across two waves of data and the domain specificity of cognitive anxieties (spatial and mathematics anxiety) as they relate to their respective skill in children. In a sample of 647 U.S. first-graders (*M_age_* = 6 years 8 months), we found bidirectional relations between spatial and mathematics skills across two waves of data collection. We found domain-specific, but not cross-domain, relations between mathematics anxiety and skills, but no evidence of links between spatial anxiety and spatial or mathematics skills at this age. These results provide support for the bidirectional spatial–mathematics link in young children and suggest that mathematics anxiety may be a useful target for interventions to improve mathematics outcomes.

## 1. Introduction

Research has consistently demonstrated that spatial cognition is linked with outcomes in science, technology, engineering, and mathematics (STEM) disciplines (e.g., Gagnier & Fisher, 2016; Shea et al., 2001; Wai et al., 2009; Wai et al., 2010). The relation between spatial and mathematics skills specifically has received more focus, and evidence for this relation has been found across development (for a meta-analysis see Atit et al., 2022). Considering these consistent findings of a moderate spatial–mathematics relation, investigating the nature of this relation is a key building block to understanding targets for intervention to improve these important skills. Some intervention and longitudinal research has found that spatial skills are causally linked with mathematics development (e.g., Bailey, 2017; Verdine et al., 2017), but less work has examined links in the opposite direction (i.e., the effect of a mathematics intervention on spatial skills or the longitudinal relation between early mathematics and later spatial skills). The longitudinal work that examines both directions has shown bidirectional relations between spatial and mathematics skills, suggesting that both spatial and mathematics skills may play a role in the development of the other (Geer et al., 2019; Lombardi et al., 2019). However, further research is needed to understand the directional nature of this relation, particularly in young children, when the development of these skills begins.

In addition, the role of domain-specific anxiety has been identified throughout the literature as a vital affective factor linked with cognitive skills in mathematics and spatial domains (Barroso et al., 2021; Geer et al., 2024). Existing work finds small-to-moderate relations between spatial and mathematics anxiety and performance in their respective domains even early in development (Ganley & McGraw, 2016; Lauer et al., 2018; Ramirez et al., 2012; Ramirez et al., 2013). However, it is unclear whether these domain-specific anxieties in young children are developed enough to play a role in skills in other domains or if they are tied to only skill in their respective domains. The domain specificity of these relations is a critical piece in best understanding how anxieties and skills in mathematics and spatial cognition intertwine across development. Given the current literature base, the present study aims to (1) extend our understanding of the longitudinal link between spatial and mathematics skills by examining potential bidirectional relations over two time points, and (2) examine the relations between spatial and mathematics anxieties and skills, with specific interest in examining if these relations are domain-specific in young children.

### 1.1. Relation Between Mathematics and Spatial Skills

Spatial skills involve a diverse group of skills that are vital to everyday life and achievement in STEM disciplines (Vasilyeva & Lourenco, 2010). Broadly, spatial skills are related to reasoning about properties of space that are captured by concepts including distance and direction (Vasilyeva & Lourenco, 2010). Although not formally taught in school settings, spatial skills are among the cognitive skills that continually develop throughout the lifespan (Harris et al., 2013; Vasilyeva & Lourenco, 2010). Furthermore, spatial skills have been shown to be malleable through a variety of intervention methods (Lerner, 2006; Lowrie et al., 2017; Uttal et al., 2013), making them a useful skill to target when improving skill in domains that rely on spatial understanding (e.g., mathematics outcomes, Hawes et al., 2017; Schmitt et al., 2018; Schmitt et al., 2025; Sorby et al., 2018).

Despite the diverse nature of the skills involved in spatial thinking, research has consistently found a close relation between a variety of spatial skills and mathematics skills across development (e.g., Burnett et al., 1979; Casey et al., 1995, 1997, 2001; Delgado & Prieto, 2004; Ganley & Vasilyeva, 2011; Geary et al., 2000; Geer et al., 2019; Reuhkala, 2001). The research literature suggests that both children and adults with better spatial skills perform better on tests of mathematics skills. While there has been some work done on the spatial–mathematics relation in younger samples (Bailey, 2017; Geer et al., 2019; Geer et al., 2025; Hawes et al., 2019; Verdine et al., 2017), there is less work at this age than with adolescents and adults. Furthermore, the precise nature of this relation, particularly the longitudinal directional nature of this relation, is still not fully understood. Examining the relation between mathematics and spatial skills over time in early elementary school students can help to tease apart the potential direction of this relation when formal mathematics learning is just beginning.

#### Theoretical Relations Between Mathematics and Spatial Skills

Theoretically, there are four possible ways in which spatial and mathematics skills may be longitudinally related. First, spatial and mathematics skills may not be directly related, but instead linked through shared variance with other cognitive processes (e.g., Hawes & Ansari, 2020; Mix & Cheng, 2012). However, research shows that the relation does seem to persist even after other cognitive variables, such as executive function, are accounted for (e.g., Atit et al., 2022; Geer et al., 2025; Hawes et al., 2019; Verdine et al., 2017). These findings suggest there may still be a unique relation between spatial and mathematics skills outside of general cognitive functioning.

Second, it may be that early spatial skills primarily impact later mathematics skills. This direction has long been the focus of much of the longitudinal and intervention research on the spatial–mathematics link (e.g., Casey et al., 2015; Gilligan et al., 2017; Gunderson et al., 2012; Lachance & Mazzocco, 2006; Lauer et al., 2018; LeFevre et al., 2013; Rutherford et al., 2018). For example, many studies provide support for the impact of training spatial skills on subsequent mathematics outcomes (e.g., Bower et al., 2020; Gilligan et al., 2020; Hawes et al., 2017; Schmitt et al., 2018; Schmitt et al., 2025). Some researchers have speculated that spatial skills, which begin to develop prior to formal schooling (e.g., Lauer & Lourenco, 2016; Ribeiro et al., 2020; Verdine et al., 2017), serve as a foundational or core component to developing mathematics skills due to overlapping skillsets and the spatial way in which mathematics is taught (i.e., the use of number lines, diagrams, etc.). Spatial skills are not formally taught but develop through exposure to the environment as well as exposure to spatial language by parents, teachers, and older siblings, which has been shown to be related to outcomes in both spatial and mathematics skills (e.g., Gilligan-Lee et al., 2021). Spatial language learned prior to formal schooling may lead children to have an easier time comprehending similar language when it is used in formal schooling to teach math. Additionally, activities such as early block play and puzzle games—which are inherently spatial tasks (blocks: Verdine et al., 2014; puzzles: Levine et al., 2012)—may be linked to concepts in mathematics such as geometry, as understanding how to visualize parts of shapes fitting together is a skill that would help students in this content area.

Conversely, the third theoretical possibility suggests that early mathematics skills may be linked to later spatial skills (e.g., Geer et al., 2019; Lombardi et al., 2019). Some concepts in mathematics are more inherently spatial in nature (i.e., number lines, geometry), but there is less work directly examining the specific mathematics skills that may be most closely related to specific subtypes of spatial skill (Gunderson et al., 2012; Hawes et al., 2019; Tam et al., 2019). If some concepts in mathematics comprise more spatial skills or even spatial language, it follows that learning these mathematics skills in a formal context may lead to improved spatial skills. Having strong mathematics skills may lead to better spatial skills because the skills learned in mathematics classes make someone think about mathematics in a more spatial way, which provides practice with thinking about space. Specifically, learning mathematics skills may lead to strengthening spatial representations of numbers as well as shapes. For example, understanding that 0 falls before 10 on the number line and understanding the proportions of the lengths of sides in an equilateral triangle might then facilitate one’s ability to visualize these numerical properties in space. This directional link may seem less intuitive than the spatial to mathematics link; however, there is research suggesting that the way students are taught to conceptualize mathematics is inherently spatial in nature (Barsalou, 2008; Gunderson et al., 2012; Mix et al., 2016); as such, it follows that formal mathematics schooling may improve spatial skill development.

The fourth theoretical possibility is that there is a bidirectional/reciprocal longitudinal relation between spatial and mathematics skills, such that early spatial skills predict later mathematics skills and early mathematics skills predict later spatial skills. This possibility encompasses the previous two possibilities and suggests that bidirectional relations may occur simultaneously and over time. Recently, some longitudinal research on the spatial–mathematics relation has considered the possibility of a bidirectional relation between spatial and mathematics skills (Bailey, 2017; Geer et al., 2019; Lombardi et al., 2019; Verdine et al., 2017). However, the results of this work are mixed. Some findings suggest that the relation between spatial and mathematics skills is, on some level, bidirectional (Geer et al., 2019; Lombardi et al., 2019), meaning that early spatial skills predict later mathematics skills and vice versa. Other work has not found evidence of a bidirectional link between these constructs (Bailey, 2017; Verdine et al., 2017). Additional work in this area focused on young children could elucidate the direction of this relation during early schooling and prove useful when aiming to develop interventions in mathematics achievement.

### 1.2. Domain-Specific Anxieties

Cognitive skills develop in tandem with affective factors, and research finds that domain-specific anxieties are often related to outcomes on various cognitive tasks. Specifically, research on attainment in mathematics has focused on how mathematics anxiety, the fear and worry related to mathematics stimuli and situations (Ashcraft, 2002; Richardson & Suinn, 1972), is a critical predictor of mathematics-related outcomes such as mathematics achievement and mathematics avoidance (e.g., Ashcraft, 2002; Beilock, 2008; Richardson & Suinn, 1972; Wigfield & Meece, 1988). Similarly, a fair amount of research has been devoted to examining the link between spatial skills and spatial anxiety, which is fear felt when engaged in spatial thinking (for a meta-analysis see Geer et al., 2024). However, much of this research, and scale development for spatial anxiety measures, has been done in adult populations (Lawton, 1994; Lyons et al., 2018; Malanchini et al., 2017). Recent interest in understanding the development of both mathematics anxiety and spatial anxiety has led to an increase in examining these anxiety types in younger populations (Dowker et al., 2016; Harari et al., 2013; Jameson, 2014; Lauer et al., 2018; Ramirez et al., 2013; Wu et al., 2012). However, there is generally less work examining these domain-specific constructs early on, likely due to the difficulties of measuring emotions and anxiety constructs in young children (Besenski et al., 2007; Ganley & McGraw, 2016; Schniering et al., 2000). These challenges include children’s limited reading and vocabulary skills and ability to understand both the concept of anxiety and the selected rating scale used for each item (Ganley & McGraw, 2016).

#### 1.2.1. Within-Domain Relations Between Cognitive Anxieties and Skills

Mathematics anxiety is an important factor related to mathematics skills (e.g., Aiken, 1970; Barroso et al., 2021; Foley et al., 2017; McLeod, 1994). Several meta-analyses investigating the relation between mathematics anxiety and mathematics achievement over the course of 30 years of research report that the overall mathematics anxiety–achievement association is statistically significant, negative, and small-to-moderate in size (Barroso et al., 2021: *r* = −0.28; Namkung et al., 2019: *r* = −0.34; J. Zhang et al., 2019: *r* = −0.32). There is less research examining this relation in young children as the research is disproportionately based on undergraduate and graduate student samples (Barroso et al., 2021). However, the available work in young children does find evidence of a significant relation between mathematics anxiety and skills (Barroso et al., 2021; Ganley & McGraw, 2016; Lauer et al., 2018).

Importantly, early development of both mathematics anxiety and mathematics achievement have separately been shown to be associated with later high school and college career interests, as well as choices in STEM fields (Ahmed, 2018; Lauermann et al., 2015; Watt et al., 2017). As such, examining how this relation unfolds over time, particularly early on in development, is important for explaining later academic and career trajectories. Available research on the longitudinal link between mathematics anxiety and mathematics skills in young children that accounts for past mathematics achievement or anxiety has shown significant, albeit weak, bidirectional relations between early mathematics anxiety and later achievement and vice versa (Gunderson et al., 2018; Szczygieł et al., 2024). These findings indicate the influential nature of both early mathematics emotions and achievement on the development of the other, even in the first few years of formal schooling.

Like the work on mathematics anxiety and mathematics skills, there is a fair amount of research dedicated to understanding the relation between spatial anxiety and spatial skills (e.g., for a meta-analysis, see Geer et al., 2024). This research suggests that due to the negative feelings associated with experiencing spatial anxiety, it is possible that some individuals with spatial anxiety will avoid tasks that involve spatial skills. This avoidance could serve to decrease opportunities to improve those skills. Spatial anxiety has been shown to relate to performance on a variety of different spatial tasks (Kremmyda et al., 2016; Lawton, 1994; Lyons et al., 2018; Ramirez et al., 2012; Sokolowski et al., 2019), as well as to higher levels of mathematics anxiety (Ferguson et al., 2015; Malanchini et al., 2017). A recent meta-analysis showed that the overall average correlation between spatial anxiety and spatial skills is statistically significant and negative, but small (Geer et al., 2024; *r* = −0.14). Importantly, there is markedly less work examining this relation in young populations (Lauer et al., 2018; Ramirez et al., 2012). However, the meta-analysis by Geer et al. (2024) did find that the spatial skill–anxiety link was weaker, though not statistically different from the overall average effect size in samples under 18 years old (*r* = −0.08). As such, the relation between spatial anxiety and spatial skills, while small, remains an important one to understand in young children.

#### 1.2.2. Domain-Specific Relations Between Cognitive Anxieties and Skills

With respect to the relations between cognitive anxieties (i.e., spatial and mathematics anxiety) and their relative cognitive skills (i.e., spatial and mathematics skills), an important consideration that emerges is the potential for domain-specific and cross-domain relations between these constructs. Domain specificity of anxiety refers to the idea that a specific type of anxiety (i.e., mathematics anxiety) is associated with, but distinct from, general anxiety (Lauer et al., 2018). Importantly, research has demonstrated that mathematics anxiety is, in fact, domain-specific across development (Hembree, 1990; Kazelskis et al., 2000; Lauer et al., 2018; Wang et al., 2014), but less work has examined the domain specificity of spatial anxiety (Daker et al., 2022; Lauer et al., 2018; Malanchini et al., 2017). This is particularly important in young children, who may not yet have developed distinct understandings of different academic domains and their own skills within them, potentially limiting their differentiation of anxiety across those domains.

In a recent study, Lauer et al. (2018) examined the relation between domain-specific anxieties (math, spatial, and verbal anxieties) and their respective cognitive skills (math, spatial, and verbal skills) in young children. Correlations demonstrated a strong link between anxieties across different domains, as well as between skills of different domains. Mathematics anxiety was significantly related to spatial skills, but spatial anxiety was not related to mathematics skills. In a regression analysis, they found that both mathematics and verbal anxieties were significant predictors of mathematics performance, but spatial anxiety was not. However, they found that none of the individual anxieties uniquely predicted spatial reasoning. As such, the results from this study suggest partial support for domain specificity, but only for mathematics skills (Lauer et al., 2018). The present study contributes to this line of work by further examining domain specificity of the relations between spatial and mathematics anxieties and skills in young children.

### 1.3. The Present Study

The overarching goal of the present study is to expand upon the existing work examining the direction of the link between spatial and mathematics skills in early elementary-aged children and to explore the relation between mathematics and spatial skills and their relative domain-specific anxieties. We asked two research questions.

Research Question 1: What is the nature of the relation between spatial and mathematics skills in first-grade students across two waves of data collection? Specifically, do we find evidence of early spatial skills predicting later mathematics skills, early mathematics skills predicting later spatial skills, or both (indicating bidirectional relations)? Based on the existing literature, we expected to find concurrent relations between spatial and mathematics skills; however, the existing findings in the literature are mixed for the direction of the longitudinal relation between mathematics and spatial skills. Therefore, we do not have one specific hypothesis for these relations. Based upon the existing research we would expect to find that either spatial skills positively predict mathematics skills, mathematics skills positively predict spatial skills, or that both are true (i.e., reciprocal positive relations).

Research Question 2: Do the relations between spatial and mathematics anxiety for children at the beginning of first grade (Wave 1) and spatial and mathematics skills at the end of first grade (Wave 2) exhibit domain specificity? Specifically, is spatial anxiety at Wave 1 more predictive of spatial skills at Wave 2 than it is of mathematics skills at Wave 2, and is the reverse true for mathematics anxiety? Based on the literature, we expected to find concurrent relations between each cognitive anxiety and their respective cognitive skill (i.e., mathematics anxiety will be correlated with mathematics skills), but it is unclear if this relation will be exclusively domain-specific. Additionally, we expected to find Wave 1 mathematics anxiety to be more predictive of mathematics skills than spatial skills, and the reverse for spatial anxiety, with spatial anxiety being more predictive of spatial skills than mathematics skills.

Across both models, we included gender and socioeconomic status as covariates, given their relations with the key variables in this study (e.g., gender: Ganley & Vasilyeva, 2011; Geary et al., 2000; Lauer et al., 2019; Levine et al., 1999; socioeconomic status: Sirin, 2005; Suna et al., 2020). We also included age to account for developmental maturation. As a robustness check, we also reran each model with a measure of reading skills in the model to see if any of the findings changed when accounting for reading skills.

## 2. Methods

The present study was preregistered following the completion of data collection for a larger study, the REALM Project. Data analysis plans were laid out prior to looking at the data. The preregistration is available at https://osf.io/ejkv7 (accessed on 26 April 2026). The present research was approved by the institutional review board at Florida State University, in accordance with the provisions of the Declaration of Helsinki.

### 2.1. Participants

The sample included 647 first-graders (*M_W1age_* = 6 years, 8 months, *SD_W1age_* = 4.7 months, range = 5 years–8 years 6 months) from 54 classroom across 25 schools in 4 counties in Florida. The data were from two waves of data collected in the fall of 2018 when children were at the beginning of first grade and the spring of 2019 when children were at the end of first grade.

We obtained information about student race/ethnicity, gender, free/reduced lunch status, parent occupation, and parent education from the students’ parents via a paper survey sent home with each student. Descriptive statistics showed that the students come from a wide variety of backgrounds. Of the 87.6% of our sample who reported free/reduced lunch status data, 49.9% qualified for free or reduced-price lunch. Of the 94.4% who reported information on their child’s race/ethnicity, 5.1% reported their children as identifying as Asian, 21.2% as Black, 12.7% as Hispanic, 48.2% as White, 0.2% as other race, and 12.6% as Multiracial. Of the 94.9% who reported their child’s gender, parents reported that roughly half of students (49.5%) identified as girls and 50.5% identified as boys.

### 2.2. Measures

#### 2.2.1. Spatial Skills

As a measure of spatial skills, we included the 16-item mental rotation subtest of Thurstone’s Primary Mental Abilities Test (Thurstone, 1938; Thurstone & Thurstone, 1949). For each item, students saw a target shape and had to figure out which of the four shape choices makes a square when aligned with the target shape. These items were given with a planned missing design, which involved dividing the 16 items into 4 subgroups of 4 items each (labeled X, A, B, C) and providing each participant with a subset of 12 items assigned by giving them three of the subgroups (e.g., Form A of the testing booklet had items from subgroups X, A, and B). Each classroom was randomly assigned to one of the three forms, meaning that students received one of three combinations of the spatial skills questions (i.e., XAB, XAC, & XBC). For each item, participants received a score of 0 for an incorrect answer and 1 for a correct answer. Test scores were determined using a two-parameter logistic model based on item-response theory using the expected a posteriori (EAP) method in MPlus (RMSEA = 0.02 and 0.03; CFI = 0.92 and 0.89, TLI = 0.90 and 0.88; marginal reliabilities were 0.57 and 0.61 for Waves 1 and 2, respectively). Full information maximum likelihood (FIML) was used to account for both the planned missing design and other item-level missing data. Higher scores indicate higher mental rotation skills, and lower scores represent lower mental rotation skills.

#### 2.2.2. Mathematics Skills

At Waves 1 and 2, students completed the Elementary Mathematics Student Assessment (EMSA; Schoen et al., 2021), which is a mathematics assessment featuring a mixture of multiple-choice and constructed-response questions in both numeric and word problem formats. The measure was designed to test mathematics knowledge and assessed the following mathematical domains: (1) counting and basic number facts, (2) word problems, and (3) number relations, fractions, and computation. The Wave 2 assessment featured some more challenging questions to account for what students would have learned throughout the school year, but some items overlapped with the Wave 1 assessment in a common-item design (Kolen & Brennan, 2004). The assessment had 22 items at each wave, though only 18 items contributed to the final scale. Test scores were determined using a two-parameter logistic model based on item response theory across the 4 grades in the full dataset (K-3). The EAP method was used to estimate each students’ skill level in each grade level. Thus, a student’s EMSA score for each wave was operationalized as a theta score, with a higher score indicating a higher level of mathematics achievement and a lower score indicating a lower level of mathematics achievement. The marginal reliability was 0.80, and more details are available in Schoen et al. (2021).

#### 2.2.3. Spatial Anxiety

As a measure of self-reported spatial anxiety, we included a modified version of the Child Spatial Anxiety Questionnaire (Ramirez et al., 2012), which included the original 8 items and 4 additional researcher-developed items (partially adapted from Lauer et al., 2018) for a total of 12 items (such as, “How do you feel when you have to solve a maze like this in one minute? [maze was shown on the same page as the item]”). Items were given to participants using a planned missing design with the same structure as the mental rotation task (i.e., XAB, XAC, and XBC). However, in this case, each participant got 9 of the 12 items. Items were answered on a pictorial scale with five answer choices (the pictures were a series of faces ranging from negative emotion to positive emotion). Confirmatory factor analysis was used to derive factor scores for each individual student to be used in analyses, with FIML used to account for both the planned missing design and other item-level missing data. McDonald’s omega for the spatial anxiety scale was 0.86, indicating good reliability.

#### 2.2.4. Mathematics Anxiety

To measure self-reported mathematics anxiety, we included an adapted version of the Mathematics Anxiety Scale for Young Children (MASYC-Revised2 Scale; Ganley et al., 2023; adapted by Ganley & McGraw, 2016; from Harari et al., 2013). This scale has 14 items rated on a Likert scale with four choices (No, Not really, Kind of, Yes). Confirmatory factor analysis was used to derive unidimensional factor scores for each individual student to be used in analyses, with FIML used to account for item-level missing data. McDonald’s omega for the mathematics anxiety scale was 0.90, indicating excellent reliability.

#### 2.2.5. Covariates

**Reading Skills:** Students completed the Test of Silent Reading Efficiency and Comprehension (TOSREC; Wagner et al., 2010), which is a standardized, group administered measure of silent reading fluency and comprehension. Students were given 3 min to read up to 50 sentences and choose whether the sentence makes sense or can be true. Scores were calculated by counting the number of items answered correctly and subtracting the number of items answered incorrectly (to account for guessing). Scores below 0 were assigned a 0. This measure has been shown to be reliable, with reliability coefficients that exceed 0.85 (Wagner et al., 2010).

Teachers were given step-by-step instructions for administration that included the 3 min limit for this task. However, some teachers did not set a time limit and therefore most or all of their students had time to complete all of the items on the assessment. This impacted 12% of students at Wave 1 and 19% at Wave 2. To account for this, we conducted a regression analysis predicting TOSREC scores from whether the teacher used a time limit or not. We then pulled the residualized scores from this analysis and standardized them and used these scores in our analyses so that the score more closely approximated what they would be if everyone received the time limit.

**Gender:** Gender was reported by parents of the participants in a basic demographics survey. It was a written response, and they were coded into boy (0), girl (1) or other (missing).

**Socioeconomic Status (SES)**: Parents were asked to report their own occupation and highest completed level of education and those of any other caregiver in the household. They also reported whether their child qualified for free or reduced-price lunch. We created a composite variable for SES by standardizing scores for occupational prestige, highest education level, and lunch status, and then calculating their average to create the composite variable. To create a composite variable, participants needed to have data on all three items; otherwise, the participants’ score was coded as missing.

***Occupational prestige***: To code parent occupations, we used the Standard International Occupational Prestige Scale (SIOPS; Treiman, 1976). This scale provides a list of broad categories of occupations (i.e., health professionals, secondary education teaching professionals, mechanics, writer, and artists) and provides a number rank associated with the prestige of the job. We coded the responses parents provided for themselves and for the additional caregiver. If only one caregiver’s occupation was listed, we only used their information for this variable. If two caregivers’ occupations were listed, we coded both of their occupations and took an average of their coded information. If the parent did not provide their occupation or if they did not provide enough information about their occupation for us to code the career, we coded these data as missing. The SIOPS does not include some jobs in their list of categories, as such, we coded specific listed occupations as missing, including if parents listed that they were disabled, a stay-at-home parent, a student, or retired.

***Level of education*:** We provided several choices for level of education (Less than 9th grade, 9th to 12th grade (no diploma), high school graduate (including equivalency/GED), some college (no degree), associate’s degree, bachelor’s degree, graduate or professional degree, or not sure). For this variable, as with occupational prestige, if the reporting parent was the only caregiver listed, we used their answer, or, if answers were provided for both the reporting parent and an additional caregiver, we used an average of the reporting parent and the additional caregiver’s level of education. Responses were coded using the values of the scale on which they answered, with 1 indicating “less than 9th grade” and 7 indicating “graduate or professional degree” (i.e., increasing values suggest higher level of education). Blank and “not sure” answers were considered missing.

***School lunch status*:** Caregivers were asked to choose their child’s lunch status from three options: free school lunch, reduced-price school lunch, or neither. These responses were coded as 0 for neither and 1 for both free and reduced-price lunch.

**Age:** We asked parents/guardians to report students’ birthdates and then calculated their age in months when they completed the Wave 1 survey (which was usually within only a few days of completing the Wave 1 mathematics test). If a student did not complete the Wave 1 survey but completed the Wave 1 mathematics test, then we calculated their age using the Wave 1 mathematics test date. When the date the Wave 1 survey was taken was not reported, we used the midpoint date of the 3-week timespan during which the teachers had the testing materials. We only included age for students who were between 60 and 102 months (started the year at age 5–8.5 years) to remove any errors in birthdate reporting. We imputed Wave 1 age from Wave 2 age when only Wave 2 was reported. It should be noted that Wave 1 age was included as a covariate after the preregistration because we wanted to account for developmental maturation that may be present regardless of the students being in the same grade.

### 2.3. Procedure

In fall 2018 (Wave 1) students completed the 30 min mathematics assessment in one testing session and the mathematics anxiety scale, spatial anxiety scale, spatial task, and reading assessment during a second 30–45 min testing session. In spring 2019 (Wave 2), students repeated the same assessments. At Waves 1 and 2, students also completed a number of other measures and assessments that are beyond the scope of this project. At both timepoints, teachers administered all measures to students in their regular classroom using materials provided by the researchers. The instructions and each test item on all included measures (except for the reading measure) were read aloud by the teacher to ensure students could comprehend the item, and students were instructed to read along with them in the provided survey booklet and then respond to the question in the space provided. Teachers received financial compensation for their participation, and students were compensated with a pencil and bookmark. Both parental consent and student written assent were required for students to be included in the study.

## 3. Results

The main analyses presented below follow our preregistered analytic plan. However, due to concerns regarding measurement error, particularly for our spatial skills measure, which were unknown at the time of preregistration, we conducted additional sensitivity analyses to test if the results held while accounting for measurement error. This included models with a latent variable using item-level data for spatial skills and models with fixed error variances based on reliability estimates for mathematics skills. Results of these sensitivity analyses are briefly summarized following our preregistered analyses below.

### 3.1. Preliminary Analyses

Descriptive statistics were calculated in SPSS Version 31.0.0.0 for all measures using derived IRT and factor scores. These descriptives are reported in Table 1. None of the results suggest significant skew or kurtosis. As per our preregistered plan for outliers, we focused on outliers that were due to error (i.e., data entry error, child response error) in these data. Based on the descriptive statistics for each variable, we were able to confirm that there were no impossible values included in these data, and thus we did not need to address any data entry errors.

Correlational results from Mplus (Muthén & Muthén, 1998–2017) can be found in Table 2. Correlations were run using the IRT and factor scores that were calculated for the main analyses. We found strong significant correlations between mathematics skills at Wave 1 and 2 (*r* = 0.66, *p* < .001) and spatial skills at Wave 1 and 2 (*r* = 0.54, *p* < .001). Additionally, there were medium-sized significant relations between mathematics and spatial skills within and across time points (*r*s = 0.33–0.39, *p*s < .001). For mathematics anxiety, we found significant small to moderate negative correlations with mathematics skills at Wave 1 (*r* = −0.25, *p* < .001) and Wave 2 (*r* = −0.27, *p* < .001). We also found significant small negative correlations between mathematics anxiety and spatial skills at Wave 1 (*r* = −0.16, *p* < .001) and Wave 2 (*r* = −0.15, *p* < .001). Mathematics anxiety and spatial anxiety were moderately correlated (*r* = 0.35, *p* < .001). Spatial anxiety was not significantly correlated with mathematics or spatial skills at either wave (*r*s = −0.07–−0.01, *p*s > .120).

### 3.2. Research Question 1

#### 3.2.1. Preregistered Analyses for Research Question 1

**Figure 1:**
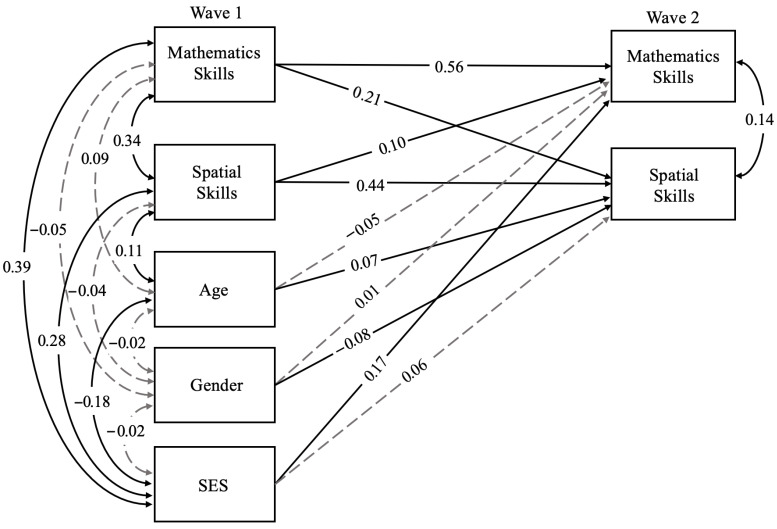
Model for Research Question 1: Relations between Spatial and Mathematics Skills over Time. For this model, black solid lines denote significant results (*p* < .05) and grey dashed lines denote non-significant results (*p* > .05). SES refers to socioeconomic status.

We first examined the relation between spatial and mathematics skills over two waves of data collection to determine if early spatial skills predict later mathematics skills, early mathematics skills predict later spatial skills, or both (indicating bidirectional relations). We estimated a cross-lagged panel model (CLPM) with spatial and mathematics skills at Wave 1 predicting spatial and mathematics skills at Wave 2, including three covariates (age, gender, and SES; see Figure 1). We accounted for nesting within classrooms by incorporating a classroom-level cluster variable in the model using type = complex in Mplus. FIML was used to account for missing data. This model allows us to address how these constructs are related concurrently and longitudinally (see Table 3 for detailed results across all included variables).

Our results indicated that mathematics and spatial skills were statistically significantly correlated within Wave 1 (*r* = 0.34, *p* < .001) and within Wave 2 (*r* = 0.14, *p* < .001). Additionally, there were statistically significant autoregressive paths between mathematics skills at Waves 1 and 2 (*β* = 0.56, *p* < .001) and spatial skills at Waves 1 and 2 (*β* = 0.44, *p* < .001). Our results also indicated bidirectional relations: Wave 1 mathematics skills significantly predicted of Wave 2 spatial skills (*β* = 0.21, *p* < .001) and Wave 1 spatial skills significantly predicted mathematics skills at Wave 2 (*β* = 0.10, *p* = .001). As preregistered, a second version of this model was run with reading included as an additional covariate as a robustness check, and the pattern of results remained consistent. See the Supplemental Materials for more detail (Figure S1).

#### 3.2.2. Exploratory Sensitivity Analyses for Research Question 1

We reran this model in two ways as an exploratory (i.e., not preregistered) sensitivity analysis, due to concerns regarding measurement error. One model was run with spatial skills modeled as a latent factor and another model was run both with spatial skills modeled as a latent factor and with mathematics skills modeled with fixed error variances based on reliability estimates. The results across both model specifications were generally consistent. Specifically, the pathway for mathematics skills at Wave 1 predicting spatial skills at Wave 2 remained significant across all iterations of the model. The pathway for spatial skills at Wave 1 predicting mathematics skills at Wave 2 remained significant in the model with only the latent variable for spatial skills (*β* = 0.16, *p* = .001) but was not significant in the model that also had mathematics skills measurement error correction (*β* = 0.11, *p* = .079). Notably, the effect size was similar to that from the preregistered analysis (*β* = 0.10), suggesting some sensitivity to measurement modeling decisions in the statistical test, but not in the size of the effect. For simplified figures representing key findings for these sensitivity analyses for research question 1, see the Supplemental Figures S3 and S4.

### 3.3. Research Question 2

#### 3.3.1. Preregistered Analyses for Research Question 2

**Figure 2:**
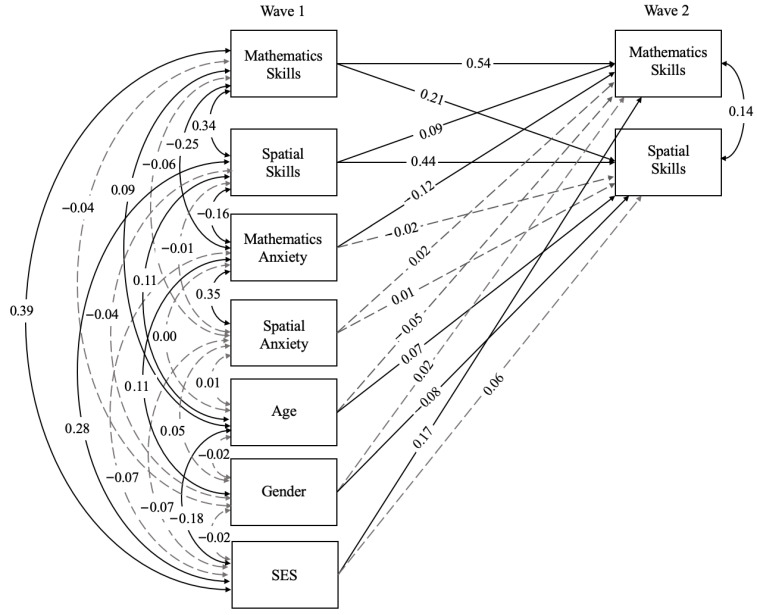
Model for Research Question 2: Relation between Cognitive Anxieties and Skills. For this model, black solid lines denote significant results (*p* < .05) and grey dashed lines denote non-significant results (*p* > .05). SES refers to socioeconomic status.

Next, we examined whether spatial and mathematics anxiety at Wave 1 predicted spatial and mathematics skills at Wave 2, with particular interest in examining whether cognitive anxieties exhibit domain-specificity (i.e., mathematics anxiety being more predictive of mathematics skills than spatial skills) in their relations with children’s cognitive performance. To address this aim, we ran the same CLPM from Research Question 1 with the addition of both spatial and mathematics anxiety at Wave 1 as predictors of Wave 2 mathematics and spatial skills, to assess whether early cognitive anxieties are predictive of later cognitive skills (see Figure 2). For detailed results across all included variables in this model, see Table 4. To address domain specificity, we conducted two Wald Tests to examine whether the domain-specific pathways (i.e., spatial anxiety → spatial skills, mathematics anxiety → mathematics skills) are statistically stronger than the cross-domain pathways (i.e., spatial anxiety → mathematics skills, mathematics anxiety → spatial skills).

The pattern of results for the relations between spatial and mathematics skills over the two waves of data collection was the same as the results from Research Question 1, showing bidirectional relations. Regarding the inclusion of cognitive anxieties in the model, we found that Wave 1 mathematics anxiety and Wave 1 spatial anxiety were significantly correlated with each other (*r* = 0.35, *p* < .001). Importantly, mathematics anxiety was significantly correlated with Wave 1 mathematics skills (*r* = −0.25, *p* < .001) and Wave 1 spatial skills (*r* = −0.16, *p* < .001). However, spatial anxiety was not significantly correlated with either Wave 1 spatial skills (*r* = −0.01, *p* = .793) or Wave 1 mathematics skills (*r* = −0.06, *p* = .163). Regarding the domain specificity of these relations, we found that Wave 1 mathematics anxiety was a significant predictor of Wave 2 mathematics skills (*β* = −0.12, *p* < .001), but it was not a statistically significant predictor of Wave 2 spatial skills (*β* = −0.02, *p* = .690). In line with the correlational results, Wave 1 spatial anxiety was not a significant predictor of Wave 2 spatial skills (*β* = 0.01, *p* = .828) or Wave 2 mathematics skills (*β* = 0.02, *p* = .641).

Results for our first Wald test showed that the domain-specific path between mathematics anxiety and mathematics skills was significantly stronger than the cross-domain path between mathematics anxiety and spatial skills (Δ*χ*^2^ = 9.13, *df* = 1, *p* = .003), suggesting domain specificity for mathematics anxiety. The second Wald Test demonstrated that there was no significant difference between the domain-specific pathway between spatial anxiety and spatial skill and the cross-domain pathway for spatial anxiety and mathematics skill (Δ*χ*^2^ = 0.08, *df* = 1, *p* = .776). A second version of this model was run with reading included as a covariate as a robustness check; the pattern of results remained consistent. See Supplemental Materials for more detail (Figure S2).

#### 3.3.2. Exploratory Sensitivity Analyses for Research Question 2

The results across sensitivity model specifications (described in Section 3.2.2) were generally consistent for research question 2, with a similar pattern of results to research question 1. The pathways for mathematics skills at Wave 1 predicting spatial skills at Wave 2 as well as mathematics anxiety at Wave 1 predicting mathematics skills at Wave 2 remained significant across all iterations of the model. The nonsignificant relations for spatial anxiety with spatial and mathematics skills remained nonsignificant. The pathway for spatial skills at Wave 1 predicting mathematics skills at Wave 2 was significant when only spatial skills was modeled as a latent variable (*β* = 0.15, *p* = .011), but became nonsignificant in the model that also included the mathematics skills measurement error correction (*β* = 0.10, *p* = .118). Though nonsignificant, the effect size was similar to that from the preregistered model (*β* = 0.09). For simplified figures representing changes to key findings for research question 2, see the Supplemental Figures S5 and S6.

## 4. Discussion

Research has routinely found both concurrent and longitudinal links between spatial and mathematics skills (e.g., Atit et al., 2022; Ganley & Vasilyeva, 2011; Gilligan et al., 2017; Mix et al., 2016). However, less work has specifically examined the potential for reciprocal longitudinal relations between spatial and mathematics skills (e.g., Geer et al., 2019; Lombardi et al., 2019; Verdine et al., 2017). Importantly, spatial skills have been shown to be trainable (Uttal et al., 2013) and have often been considered a potential target for interventions aimed to improve mathematics skills (e.g., Gilligan et al., 2020; Hawes et al., 2017; Schmitt et al., 2018; Schmitt et al., 2025). To better understand the nature of their relation over time, we examined the potential bidirectional relations between spatial and mathematics skills in a sample of first-grade students over a school year and tested whether any relations remained when accounting for reading skills.

In addition, research shows a relation between mathematics anxiety and skills (e.g., Barroso et al., 2021) and spatial anxiety and skills (e.g., Geer et al., 2024) even among younger children (e.g., Ganley & McGraw, 2016; Gunderson et al., 2018; Lauer et al., 2018; Ramirez et al., 2012). The domain specificity of these relations remains unclear (i.e., it is unclear if spatial anxiety is more closely related to spatial skills than it is to other cognitive skills in children; Lauer et al., 2018). As such, we investigated the relations between spatial and mathematics anxieties and spatial and mathematics skills and tested whether mathematics anxiety was more closely related to mathematics skills than it was to spatial skills, and whether spatial anxiety was more closely related to spatial skills than it was to mathematics skills.

Our findings provide evidence for bidirectional relations between spatial and mathematics skills such that early spatial skills predict later mathematics skills *and* early mathematics skills predict later spatial skills. In line with existing research, our results also provide evidence for a domain-specific longitudinal relation between mathematics anxiety and mathematics skills; however, spatial anxiety did not significantly predict either spatial or mathematics skills. Thus, the present study finds no evidence for domain-specific longitudinal relations between spatial anxiety and spatial skills in a sample of children in the first grade. All results remained the same when including reading skills as a covariate, suggesting that these patterns of results cannot be solely attributed to the function of general cognitive skills. In sensitivity analyses, all results held when spatial skill was modeled as a latent variable, but the relation between Wave 1 spatial skills and Wave 2 mathematics skills became nonsignificant (*p*s = .079, .118) when measurement error correction was also applied to the mathematics skills assessment despite relatively stable effect sizes across models. These findings suggest consistency in the magnitude of the relations, but some sensitivity to modeling decisions. It should be noted that throughout this paper we have used the term spatial skills to align with the overall literature, but our measure specifically assessed mental rotation, and findings related to spatial skills should be interpreted with this in mind.

### 4.1. Relation Between Spatial and Mathematics Skills

First, we examined the relations between spatial and mathematics skills over two waves of data collection to assess whether early spatial skills predict later mathematics skills, early mathematics skills predict later spatial skills, or if both occur simultaneously indicating bidirectional relations (Research Question 1). We found positive statistically significant correlations between spatial and mathematics skills within and across waves of data collection, in line with existing research (e.g., Atit et al., 2022; Bailey, 2017; Ganley & Vasilyeva, 2011; Geer et al., 2019; Lombardi et al., 2019; Mix et al., 2016; Verdine et al., 2017). Specifically, our results showed moderate positive correlations between spatial and mathematics skills, such that higher levels of spatial skill were associated with higher levels of mathematics skills both within and across time points.

With respect to the relations between spatial and mathematics skills over time, we found reciprocal relations between mathematics and spatial skills. This aligns with some of the existing research on this relation (Geer et al., 2019; Lombardi et al., 2019), though other work has found conflicting evidence on the exact nature of the relation between spatial and mathematics skills (Bailey, 2017; Verdine et al., 2017). These findings may prove important when considering possibilities for interventions to improve mathematics outcomes. The existing literature has often focused on early spatial skills impacting later mathematics skills, and interventions have often been directed at improving mathematics skills through improvement in spatial skills (e.g., Bower et al., 2020; Gilligan et al., 2020; Hawes et al., 2017; Schmitt et al., 2018; Schmitt et al., 2025; Sorby et al., 2018). Our findings converge with this past research suggesting that interventions on spatial skills may indeed improve mathematics skills; however, we may also be able to improve spatial skills through the ways in which we teach math, or we may also aim to teach mathematics in a way that is more spatial in nature such that we improve both mathematics and spatial skills. Research should continue to tease apart the mechanisms underlying the links between these factors and how these may come into play when intervening on spatial or mathematics outcomes.

We also found that these bidirectional relations remained significant when accounting for reading skills. Our results contrast with some theoretical accounts suggesting that the link between spatial and mathematics skills may rely on shared underlying cognitive resources required to complete these tasks rather than a unique relation between the two skills (e.g., Hawes & Ansari, 2020; Mix & Cheng, 2012). Our finding is in line with existing research suggesting that the spatial–mathematics link exists above and beyond measures of English/Language Arts skills, vocabulary skills, and/or executive function (Atit et al., 2022; Geer et al., 2025; Rutherford et al., 2018; Verdine et al., 2017). Our results confirm the value of the spatial–mathematics link and support the use of spatial skills as a target for interventions in mathematics (e.g., Bower et al., 2020; Gilligan et al., 2020; Hawes et al., 2017; Schmitt et al., 2018; Schmitt et al., 2025; Sorby et al., 2018).

### 4.2. Domain-Specificity of the Relations Between Cognitive Anxieties and Skills

The second aim of this study (Research Question 2) was to identify whether spatial and mathematics anxiety at Wave 1 predict spatial and mathematics skills at Wave 2 and to examine whether cognitive anxieties exhibit domain-specificity (i.e., mathematics anxiety being more predictive of mathematics skills than spatial skills) in their relations with children’s cognitive performance. We found a significant moderate negative correlation between mathematics anxiety and mathematics skills such that higher levels of mathematics anxiety are associated with lower levels of mathematics skills, in line with existing research on this relation at this age (e.g., Barroso et al., 2021; Namkung et al., 2019; J. Zhang et al., 2019). Interestingly, and contrary to expectations, there were no significant correlations between spatial anxiety and spatial skills at either wave of data collection.

We found that mathematics anxiety at the beginning of first grade (Wave 1) is a significant predictor of mathematics skills at the end of first grade (Wave 2), even when accounting for past levels of mathematics skills, in line with similar existing research in children (Gunderson et al., 2018). We did not find a parallel relation for mathematics anxiety and spatial skills, and results of the Wald Test, which allowed for direct comparisons between the strength of the relation between mathematics anxiety and later mathematics skills to the relation of mathematics anxiety and later spatial skills, confirmed that the domain-specific pathway was a stronger link than the cross-domain pathway. This finding is in line with Lauer et al. (2018), who found domain-specificity in predictive relations between mathematics anxiety and mathematics skills at this age. Thus, even as early as first grade, mathematics anxiety is a significant predictor for mathematics outcomes even when accounting for past levels of mathematics skills (Gunderson et al., 2018; Pelegrina et al., 2025) and should, therefore, continue to be considered as a potential target for interventions aimed at improving mathematics skills. These results suggest that even in first grade, mathematics anxiety is meaningfully capturing something specific to mathematics, rather than reflecting a general anxiety response. Supporting this interpretation, math anxiety was more strongly correlated with mathematics skills than with both reading and spatial skills, providing evidence for some differentiation of mathematics anxiety from both other academic domains.

Contrary to our hypotheses, however, we did not find spatial anxiety to be a significant predictor of later spatial or mathematics skills. Importantly, we did not even find significant zero-order correlations between our measures of spatial anxiety and spatial skills, in contrast with some work in this age group (Lauer et al., 2018; Ramirez et al., 2012). This finding is in line with findings from a recent meta-analysis that demonstrate a weak relation between spatial anxiety and spatial skills in samples under the age of 18 years old (*r* = −0.08; Geer et al., 2024), though our results were even weaker. We also did not find a significant relation between spatial anxiety and later mathematics skills. Results of the Wald Test comparing the domain-specific and cross-domain pathways suggest that for spatial anxiety, there was no statistically significant difference in the strengths of these nonsignificant paths. This finding is in line with those of Lauer et al. (2018), who did not find domain-specificity of predictive relations between spatial anxiety and spatial skills at this age.

It is possible, at this age, that we are not seeing a significant relation between spatial anxiety and spatial skills due to the general lack of experience children have with spatial tasks. It should also be noted that the mental rotation measure had poor reliability, which can attenuate correlations. Importantly, our measure of spatial anxiety was significantly associated with mathematics anxiety, suggesting that it is tapping into a type of cognitive anxiety. It is possible that this measure of spatial anxiety is not related to mental rotation skills (the measure of spatial skills we used) or that the wording of the spatial anxiety items was too task-specific (i.e., focused on particular spatial tasks), compared to the mathematics anxiety items, which simply ask about “math”. It is unclear whether spatial anxiety becomes more differentiated or simply emerges later in childhood; additional attention to measurement of both spatial anxiety and skills will be important for illuminating this.

When we included reading skills as a robustness check, we found that the inclusion of reading skills did not impact the significance of the relations between mathematics and spatial anxieties and their respective skills. Importantly, reading skills were significantly associated with fall mathematics and spatial skills and mathematics anxiety, suggesting that there may be some shared cognition involved, but this did not impact the statistical significance of the relations between the spatial and mathematics anxieties and mathematics or spatial skills. Our direct comparisons of the strength of the domain-specific pathways to the cross-domain pathways for mathematics and spatial anxieties and skills were also not impacted by the presence of reading skills. Thus, relations between spatial and mathematics anxiety and skills do not appear to rely on shared cognitive resources. Unfortunately, we did not have measures of reading or general anxiety in our study, but the inclusion of these in future research would help to further understand the domain generality and specificity of these relations.

### 4.3. Limitations

One limitation of the present study is that the spatial skills measure had poor reliability. The marginal reliability did improve between Waves 1 and 2, suggesting that it may not be age-appropriate for the early first-graders. However, another recent study also had issues with reliability for this measure in a sample of children spanning first through fourth grades, finding McDonald’s omegas between 0.51 and 0.64 (X. Zhang & Gunderson, 2025); therefore, it may not be strictly age-related. X. Zhang and Gunderson suggest that, perhaps, this task may measure more than one latent factor, due to the various strategies that children can use to solve the items. They suggest, for example, that children may use strategies that are more analytical in nature (e.g., mentally labelling and/or matching spatial features) or visuospatial in nature (e.g., mental rotation) to complete this task. A task that engages various strategies differs from one that purely assesses mental rotation skill, which would typically be designed to prevent analytical strategies. Regardless, future work should aim to replicate the present work with a more reliable measure of mental rotation skills.

We also only had one spatial measure, and it only measured one type of spatial skill (i.e., mental rotation), but there are many spatial skill measures and subtypes (e.g., Linn & Petersen, 1985; Uttal et al., 2013). Future work should look to replicate these findings with other types of spatial skills, as much of the existing research has centered around mental rotation (e.g., Casey et al., 1997, 1995; Ganley & Vasilyeva, 2011; Reuhkala, 2001) and thus we do not know as much about how mathematics or anxieties relate to other types of spatial skills, particularly in children. Additionally, while our measure of spatial skills was limited to mental rotation, our measure of spatial anxiety covered mental rotation (4 items) and other subtypes of spatial skills (8 items); therefore, it is possible that this mismatch in skill subtype was partially to blame for the lack of relations between spatial anxiety and spatial skills in this sample (Geer et al., 2024). Future research is needed with additional measures of spatial anxiety and skills to determine if this is the case.

Further, we did not include a measure of general cognitive ability, such as an IQ test, which limits the conclusions we can make about the influence of general cognitive factors on the relation between spatial and mathematics skills and anxieties. Future work could include general cognitive skill alongside reading skills in similar models. We also only examined the relation between these constructs in one age group. Future research should explore these relations over longer periods of time and/or in other age groups. Specifically, it would be interesting to examine the development of domain-specific anxieties (such as mathematics and spatial anxiety) across more ages to see if the relations between cognitive anxieties and their respective cognitive skills grow in strength over time as children further differentiate these different skills and anxieties.

Additionally, theta scores for spatial and mathematics skills for our preregistered analyses were estimated using EAP scoring in the IRT models. This estimator can often underestimate the true latent variance, which could have attenuated the strength of the relations among variables. While we acknowledge this as a limitation, the EAP scores for the mathematics task were pre-created as part of the larger study and are used across all manuscripts with these data (e.g., Barroso et al., 2023), as recommended by the test developer (Schoen et al., 2021). Therefore, in an effort to maintain consistency across measures in this study, we felt it prudent to use EAP for the spatial skills measure as well. As such, our results should be interpreted with the caveat that the use of EAP scores for our IRT models could reduce the magnitude of the relations we are examining. We did also conduct exploratory sensitivity analyses with a latent factor for spatial skills and measurement error correction for our mathematics skill measure. The results of these models suggest the pathway for spatial skills at Wave 1 predicting mathematics skills at Wave 2 may be somewhat sensitive to modeling decisions, though the effect sizes were almost identical to those from the preregistered analyses. It should be noted, however, that this pathway is well-established in the literature, suggesting that the instability of this finding is likely due to measurement error rather than truly null results. Taken together, our findings highlight the need for replication with more reliable spatial measures.

Lastly, the present study is limited by only including two waves of data collection within a single year of school. This substantially reduced the modeling options we had for looking at bidirectional relations, removing more methodologically sophisticated approaches such as bivariate latent change score modeling and random intercept cross-lagged panel models (RI-CLPMs) as options. Of note, an additional wave of data collection was originally planned for the larger project that data from this study were from, but the plans were unfortunately disrupted due to the COVID-19 pandemic. We feel it is vital that we acknowledge and address the potential limitations of CLPMs. First, CLPMs come with several assumptions (Kearney, 2017). While we feel confident that our data met the majority of these assumptions, we want to highlight the ones that our data may leave unaddressed. First, CLPM assumes that variables were measured without error, which is usually handled by using latent variables. Although we present sensitivity analyses with some of measurement error accounted for in some of the measures through corrections and latent variables, we did not incorporate latent variables in our main study analyses; therefore, there is a chance that our work does not meet this assumption. However, we did incorporate IRTs and CFAs to derive variables to help meet this assumption. Second, CLPM assumes that all possible variables were measured and included in the model (in theory). For the present study, we included covariates and measures that we felt were important based upon existing research, but there are likely still omitted variables (as is typical of most research). Lastly, CLPM assumes that there are no inter-participant differences over time in the stability of measures from time point 1 to time point 2, or more specifically, that unobserved differences may impact or bias results. The present work does not account for all potential interparticipant differences; however, future work could address this limitation by collecting three waves of data and conducting RI-CLPMs or bivariate latent change score models.

### 4.4. Conclusions

Our research findings show reciprocal relations between spatial and mathematics skills even when accounting for reading skills and cognitive anxieties (spatial and mathematics anxiety), suggesting that these skills may influence one another over time and could, therefore, be used as the targets for intervention when aiming to improve skills in the other. This is important because some work has suggested that the link between spatial and mathematics skills is based upon the shared cognitive processes that underly these skills, and the present findings suggest that the relation between spatial and mathematics skills exists even when accounting for another cognitive skill (reading skill) and two cognitive anxieties (spatial and mathematics anxiety). We also found evidence that mathematics anxiety is a stronger predictor of later mathematics skills than it is of later spatial skills at this age (Lauer et al., 2018). However, we did not find evidence that spatial anxiety is related to spatial or mathematics skills at this age, though these conclusions should be interpreted in the context of our spatial skills measure having fairly low reliability.

These results, in line with existing research, suggest that mathematics anxiety and spatial skills may be more useful than spatial anxiety as targets for interventions focused on improving mathematics skills, and that improving mathematics skills could also help potentially improve spatial skills. Fortunately, research does support the malleability of spatial skills (Lerner, 2006; Lowrie et al., 2017; Uttal et al., 2013), mathematics anxiety (Codding et al., 2023; Liu et al., 2025), and mathematics skills (Hinton et al., 2013), and some research has found that interventions focused on improving children’s spatial or mathematics skills or reducing their mathematics anxiety can be delivered by parents at home (e.g., bedtime mathematics application, Berkowitz et al., 2015) or educators in primary school classrooms in the early grades (e.g., block building activities, Casey et al., 2008; Schmitt et al., 2025). There is little work on mathematics skills interventions improving spatial skills, but research suggests we may see transfer effects from spatial skills intervention to mathematics learning (e.g., Bower et al., 2020; Gilligan et al., 2020; Hawes et al., 2017; Schmitt et al., 2018; Sorby et al., 2018), and mathematics anxiety intervention to mathematics performance (Codding et al., 2023; Liu et al., 2025). Thus, targeting both of these is likely to improve both mathematics and spatial skills.

## Figures and Tables

**Table 1:** Descriptive Statistics for All Included Measures.

Measure	N	Min	Max	Mean	SD	Skew	Skew S.E.	Kurtosis	Kurtosis S.E.
SES	423	−1.64	1.57	0.15	0.79	−0.30	0.12	−0.93	0.24
Mathematics Skills W1	581	−3.43	0.85	−1.33	0.90	0.26	0.10	−0.56	0.20
Mathematics Skills W2	567	−2.75	1.93	−0.04	0.89	−0.11	0.10	−0.44	0.21
Spatial Skills W1	574	−1.89	1.71	−0.01	0.73	−0.03	0.10	−0.61	0.20
Spatial Skills W2	543	−2.14	1.63	−0.02	0.76	−0.07	0.11	−0.32	0.21
Mathematics Anxiety	581	−0.67	0.97	0.01	0.37	0.18	0.10	−0.14	0.20
Spatial Anxiety	578	−0.91	1.37	0.00	0.46	0.05	0.10	−0.14	0.20
Reading W1	579	−2.43	3.12	−0.01	1.00	0.39	0.10	0.04	0.20

Notes. SES is socioeconomic status. S.E. is standard error; W1 refers to Wave 1, W2 refers to Wave 2.

**Table 2:** Zero-Order Correlations Between All Included Measures.

	Gender	SES	Age W1	Mathematics Skills W1	Mathematics Skills W2	Spatial Skills W1	Spatial Skills W2	Mathematics Anxiety	Spatial Anxiety
Gender									
SES	−0.01 (0.04)								
Age	−0.02 (0.05)	0.18 ** (0.05)							
Mathematics Skills W1	−0.05 (0.04)	0.39 *** (0.04)	0.09 (0.05)						
Mathematics Skills W2	−0.02 (0.05)	0.42 *** (0.05)	−0.02 (0.05)	0.66 *** (0.03)					
Spatial Skills W1	−0.04 (0.04)	0.28 *** (0.04)	0.11 * (0.05)	0.35 *** (0.04)	0.33 *** (0.04)				
Spatial Skills W2	−0.11 * (0.05)	0.25 *** (0.05)	0.13 ** (0.04)	0.39 *** (0.05)	0.39 *** (0.03)	0.54 *** (0.03)			
Mathematics Anxiety	0.11 * (0.04)	−0.07 (0.05)	−0.00 (0.05)	−0.25 *** (0.04)	−0.27 *** (0.04)	−0.16 *** (0.03)	−0.15 *** (0.04)		
Spatial Anxiety	0.05 (0.04)	−0.07 (0.05)	0.01 (0.05)	−0.06 (0.04)	−0.07 (0.04)	−0.01 (0.04)	−0.02 (0.04)	0.35 *** (0.04)	
Reading	0.01 (0.04)	0.24 *** (0.06)	−0.01 (0.04)	0.50 *** (0.04)	0.37 *** (0.04)	0.17 *** (0.04)	0.20 *** (0.05)	−0.14 ** (0.05)	−0.01 (0.05)

Notes. * refers to *p* < .05, ** refers to *p* < .01, *** refers to *p* < .001; W1 refers to Wave 1, W2 refers to Wave 2. Numbers within parentheses refer to the standard errors of each correlation.

**Table 3:** Results of the Cross-Lagged Panel Model for Research Question 1.

	*b (se)*	*Beta/r (se)*	*p*
Autoregressive paths			
Mathematics Skills W1 → Mathematics Skills W2	0.55 (0.03)	0.56 (0.03)	<.001
Spatial Skills W1 → Spatial Skills W2	0.46 (0.04)	0.44 (0.04)	<.001
Cross-lagged paths			
Mathematics Skills W1 → Spatial Skills W2	0.18 (0.04)	0.21 (0.05)	<.001
Spatial Skills W1 → Mathematics Skills W2	0.12 (0.04)	0.10 (0.03)	.001
Wave 1 correlations			
Mathematics Skills W1 ↔ Spatial Skills W1	0.23 (0.03)	0.34 (0.04)	<.001
Gender ↔ Mathematics Skills W1	−0.02 (0.02)	−0.05 (0.04)	.301
SES ↔ Mathematics Skills W1	0.28 (0.03)	0.39 (0.04)	<.001
Age ↔ Mathematics Skills W1	0.37 (0.22)	0.09 (0.05)	.088
Gender ↔ Spatial Skills W1	−0.02 (0.02)	−0.04 (0.04)	.312
SES ↔ Spatial Skills W1	0.16 (0.03)	0.28 (0.04)	<.001
Age ↔ Spatial Skills W1	0.39 (0.17)	0.11 (0.05)	.020
Gender ↔ SES	−0.01 (0.02)	−0.02 (0.04)	.656
Gender ↔ Age	−0.04 (0.11)	−0.02 (0.05)	.688
SES ↔ Age	−0.67 (0.19)	−0.18 (0.05)	<.001
Wave 2 correlations			
Mathematics Skills W2 ↔ Spatial Skills W2	0.06 (0.02)	0.14 (0.04)	<.001
Covariate relations for Wave 2			
Gender → Mathematics Skills W2	0.02 (0.06)	0.01 (0.03)	.770
SES → Mathematics Skills W2	0.19 (0.05)	0.17 (0.04)	<.001
Age → Mathematics Skills W2	−0.01 (0.01)	−0.05 (0.04)	.163
Gender → Spatial Skills W2	−0.13 (0.06)	−0.08 (0.04)	.030
SES → Spatial Skills W2	0.05 (0.05)	0.06 (0.05)	.308
Age → Spatial Skills W2	0.01 (0.01)	0.07 (0.04)	.043

Notes. For predictive paths, the reported statistics are unstandardized and standardized beta values, respectively; for correlational paths, the reported statistics are covariances and correlation coefficients, respectively.

**Table 4:** Results of the Cross-Lagged Panel Model for Research Question 2.

	*b (se)*	*Beta/r (se)*	*p*
Autoregressive paths			
Mathematics Skills W1 → Mathematics Skills W2	0.53 (0.03)	0.54 (0.03)	<.001
Spatial Skills W1 → Spatial Skills W2	0.45 (0.04)	0.44 (0.04)	<.001
Cross-lagged paths			
Mathematics Skills W1 → Spatial Skills W2	0.17 (0.04)	0.21 (0.05)	<.001
Spatial Skills W1 → Mathematics Skills W2	0.11 (0.04)	0.09 (0.03)	.005
Anxiety paths			
Mathematics Anxiety → Mathematics Skills W2	−0.30 (0.07)	−0.12 (0.03)	<.001
Spatial Anxiety → Mathematics Skills W2	0.03 (0.07)	0.02 (0.04)	.641
Mathematics Anxiety → Spatial Skills W2	−0.04 (0.09)	−0.02 (0.04)	.690
Spatial Anxiety → Spatial Skills W2	0.01 (0.06)	0.01 (0.04)	.828
Wave 1 correlations			
Mathematics Skills W1 ↔ Spatial Skills W1	0.23 (0.03)	0.34 (0.04)	<.001
Gender ↔ Mathematics Skills W1	−0.02 (0.02)	−0.04 (0.04)	.311
SES ↔ Mathematics Skills W1	0.28 (0.03)	0.39 (0.04)	<.001
Age ↔ Mathematics Skills W1	0.38 (0.22)	0.09 (0.05)	.080
Mathematics Anxiety W1 ↔ Mathematics Skills W1	−0.08 (0.02)	−0.25 (0.04)	<.001
Spatial Anxiety W1 ↔ Mathematics Skills W1	−0.02 (0.02)	−0.06 (0.04)	.163
Gender ↔ Spatial Skills W1	−0.02 (0.02)	−0.04 (0.04)	.284
SES ↔ Spatial Skills W1	0.16 (0.03)	0.28 (0.04)	<.001
Age ↔ Spatial Skills W1	0.39 (0.17)	0.11 (0.05)	.020
Mathematics Anxiety W1 ↔ Spatial Skills W1	−0.04 (0.01)	−0.16 (0.03)	<.001
Spatial Anxiety W1 ↔ Spatial Skills W1	0.00 (0.01)	−0.01 (0.04)	.793
Mathematics Anxiety W1 ↔ Spatial Anxiety W1	0.06 (0.01)	0.35 (0.04)	<.001
Mathematics Anxiety W1 ↔ Gender	0.02 (0.01)	0.11 (0.04)	.012
Mathematics Anxiety W1 ↔ SES	−0.02 (0.02)	−0.07 (0.05)	.142
Mathematics Anxiety W1 ↔ Age	−0.01 (0.08)	0.00 (0.05)	.933
Spatial Anxiety W1 ↔ Gender	0.01 (0.01)	0.05 (0.04)	.296
Spatial Anxiety W1 ↔ SES	−0.02 (0.02)	−0.07 (0.05)	.195
Spatial Anxiety W1 ↔ Age	0.02 (0.10)	0.01 (0.05)	.875
Gender ↔ SES	−0.01 (0.02)	−0.02 (0.04)	.706
Gender ↔ Age	−0.04 (0.11)	−0.02 (0.05)	.697
SES ↔ Age	−0.67 (0.19)	−0.18 (0.05)	<.001
Wave 2 correlations			
Mathematics Skills W2 ↔ Spatial Skills W2	0.05 (0.01)	0.14 (0.04)	<.001
Covariate relations for Wave 2			
Gender → Mathematics Skills W2	0.04 (0.05)	0.02 (0.03)	.504
SES → Mathematics Skills W2	0.19 (0.05)	0.17 (0.04)	<.001
Age → Mathematics Skills W2	−0.01 (0.01)	−0.05 (0.04)	.210
Gender → Spatial Skills W2	−0.13 (0.06)	−0.08 (0.04)	.033
SES → Spatial Skills W2	0.06 (0.05)	0.06 (0.05)	.292
Age → Spatial Skills W2	0.01 (0.01)	0.07 (0.04)	.042

Notes. For predictive paths, the reported statistics are unstandardized and standardized beta values, respectively; for correlational paths, the reported statistics are covariances and correlation coefficients, respectively.

## Data Availability

The data and a data dictionary for this study can be found here: https://osf.io/ygs67/ (accessed on 26 April 2026).

## Supplementary Materials

The following supporting information can be downloaded at https://www.mdpi.com/article/10.3390/jintelligence14050087/s1.
