# Supplementary material for: Bidirectional Relations Between Spatial and Mathematics Skills in Elementary School Children: The Role of Domain-Specific Anxieties
Source: J Intell. 2026 May 19;14(5):87. doi: 10.3390/jintelligence14050087 (PMC13207808; doi:10.3390/jintelligence14050087)
Supplement: Supplementary file 1 [file jintelligence-14-00087-s001.zip › OSF_Spatial-Math and Domain-Specific Anxieties OSF Preregistration 5-19-2021.pdf]

Part 1: General Data Information

1. **Name and briefly describe the funding sources and general sample characteristics of the dataset(s) if known.**

Data are from a larger [redacted] funded grant [redacted]. This project is investigating teachers' math anxiety and instructional practice in relation to elementary-school students' math attitudes and achievement. The first year of the grant (Cohort 1; [redacted]) consisted of a pilot year where 25 K-3 teachers and their students were recruited to participate in the study. During the second year of the grant (Cohort 2; [redacted]), 227 K-3 teachers and their students were recruited to participate in the study.

2. **Describe, to the best of your ability, how the data were collected.**

Teachers completed surveys online during early fall and then were provided with student testing packets in the fall (August- September) and were asked to administer surveys and a math test for Wave 1 of the study's student data collection. Packets were picked up after fall data collection. In late spring in the same academic year (April-May), teachers were again provided with student packets and were asked to administer surveys and tests to their students for Wave 2 of the study's student data collection. These packets were again picked up after teachers administered these tests.

3. **Some studies offer codebooks to describe their data. If such a codebook is publicly available, link to it here or upload the document.**

A codebook of the variables in the data set used for the present project will be available as a downloadable file on the preregistration website prior to the final paper being submitted for publication.

Part 2: Study and Variable Information

The current project only uses data from the first graders in the sample: approximately 600 first-grade students.

1. **Name and briefly describe the subset(s) of the data you plan to use.**

For this study, we will use data from first-grade student participants from Cohort 2. There were approximately 600 students.

2. **List each research question included in this study.**

Research Question 1: What is the magnitude and direction of the relation between spatial and math skills in young children over two waves of data collection? Does the relation between these constructs differ when accounting for another cognitive skill (reading skills)?

Research Question 2: Do the relations between spatial and math anxiety at Wave 1 and spatial and math skills at Wave 2 exhibit domain specificity? For example, is spatial anxiety at Wave 1 more predictive of spatial skills at Wave 2 than it is of math skills at Wave 2? Is the reverse true for math anxiety?

3. **Identify and describe any measured variables you will use in your study.**

Student measures

Math assessment. In fall 2018 and spring 2019, students completed a fall and spring version of the first grade 22-item Elementary Mathematics Student Assessments (EMSA) during the fall and spring, respectively (adapted from the fall 2015 [Schoen et al., 2018a] and spring 2016 [Schoen et al., 2018b] versions of the EMSA). The measure was designed to test math knowledge and assessed the following mathematical domains: 1) counting and basic number facts, 2) word problems, and 3) number relations, fractions, and computation. Some items were presented in a selected response format and some items were presented in a constructed response format. The EMSA score was operationalized as a theta score, with a higher score indicating a higher level of math achievement and a lower score indicating a lower level of math achievement. Any students who did not answer 30% or more of the items on the EMSA will not receive a theta score.

- **Psychometric properties**. Detailed psychometric information about this measure will be available in technical reports for the Fall 2018 and Spring 2019 EMSA. Reliability information will be reported in the final paper.

Spatial skills task. In fall 2018 and spring 2019, students completed a measure of mental rotation skill: the mental rotation subtest of Thurstone's Primary Mental Abilities Test (Thurstone, 1974). For each item, students saw a target shape and had to figure out which of the four choice shapes makes a square when aligned with the target shape. This task

consists of 16 items. These items were given with a planned missing design, which involved dividing the 16 items into four subgroups of 4 items each (labeled X, A, B, C) and providing each participant with a subset of 12 items assigned by giving them three of the subgroups (i.e., Form A of the testing booklet had items from X, A, and B). Each student was randomly assigned to one of 3 forms for the assessments, meaning that students received one of three combinations of the spatial anxiety questions (i.e., XAB, XAC, & XBC). For each item, participants received a score of 0 for an incorrect answer and 1 for a correct answer. We will derive an IRT score for this task for each participant.

- **Psychometric properties**. Reliability information, such as Cronbach's alphas will be reported in the final paper.

Math anxiety. We measured math anxiety using an adapted version of the Math Anxiety Scale for Young Children (MASYC-Revised Scale; adapted by Ganley & McGraw, 2016; from Harari et al., 2013). This scale has 14 items rated on a Likert scale with four choices (no, not really, kind of, yes). These items fall across three subscales: negative reactions (5 items; i.e., When it is time for math my head hurts), numerical confidence (3 items; i.e., Doing math problems on the board in front of the class makes me nervous.), and worry (6 items; i.e., I get nervous about making a mistake in math.).

- **Psychometric properties**. Extensive reliability and validity information is provided in Ganley and McGraw (2016) for an earlier version of this scale. Reliability information, including item-total correlations and Cronbach's alphas will be reported in the final paper for this updated version of the scale.

Spatial anxiety. We measured spatial anxiety using a modified version of the Child Spatial Anxiety Questionnaire (Ramirez et al., 2012) which included the original 8 items as well as 4 additional researcher-developed items (partially adapted from Lauer et al., 2018) for a total of 12 items (such as, "How do you feel when you have to solve a maze like this in one minute? [Show picture with maze]"). Items were given to participants using a planned missing design with the same structure as the mental rotation task (i.e., XAB, XAC, and XBC). However, in this case, each participant got 8 of the 12 items. These 8 items were answered on a pictorial scale with five answer choices (the pictures were a series of faces ranging from negative emotion to positive emotion).

- **Psychometric properties**. Reliability information, including item-total correlations and Cronbach's alphas will be reported in the final paper.

Reading skills. In fall 2018, students completed a measure of reading comprehension skill: The Test of Silent Reading Efficiency and Comprehension (TOSREC; Wagner et al., 2010), which is a standardized, group administered measure of silent reading fluency and comprehension. Students were given 3 minutes to read sentences and choose whether each sentence makes sense or can be true. Scores were calculated by counting the number of items marked correct and subtracting the number of items marked incorrect (to account for guessing).

Gender. Parents/guardians were asked to provide their child's gender. Student gender was coded as girl (coded as 0), boy (coded as 1), or blank (coded as missing) if parents did not provide this information.

Socioeconomic status (SES). Parents were asked to report their own occupation and highest level of education completed as well as this information for any other additional caregiver of the child. They also reported whether their child qualifies for free or reduced-price lunch. We operationalized SES as a manifest variable that was calculated by a combination of occupational prestige, highest education level, and lunch status. Each of the three items were coded (coding detailed below), standardized, and then combined to create the variable. To create a composite variable, participants had to have data on all three items; otherwise, the participants' SES score was left blank.

- *Occupational prestige*. We used the Standard International Occupational Prestige Scale (SIOPS; Treiman, 1976) to code parent occupations. This scale consists of a list of broad categories of occupations, such as health professionals, secondary education teaching professionals, mechanics, writer, and artists. We coded the responses parents provided for the primary caregiver (i.e., person filling out form) and for the additional caregiver if that caregiver was also a parent/guardian using the coding scheme from the SIOPS as our measure of occupational prestige. If only one parent/guardians' occupation is listed, we only used their information for the occupational prestige variable. If both caregivers' occupations are listed and the second caregiver is also a parent/guardian, we coded both of their occupations, and an average of their coded information will be used for the occupational prestige variable.

  If the parent did not provide their occupation or if they did not provide enough information about their occupation for us to code the career, we coded these data as missing. Because the SIOPS does not include some jobs in their list of categories, we coded specific listed occupations as missing, including if parents listed that they were disabled, a stay-at-home parent, student, or retired.

- *Level of education*. Choices for level of education were: Less than 9th grade, 9th to 12th grade (no diploma), high school graduate (including equivalency/GED), some college (no degree), associate degree, bachelor's degree, graduate or professional degree, or not sure. For the highest level of education variable, we will use the data reported by the primary caregiver if their information is the only one available, or an average of the primary caregiver's and the secondary caregiver's level of education if the secondary caregiver is identified as another parent/guardian. Parent responses will be coded using the categories of the scale, with 1 indicating "less than 9th grade" and 7 indicating "graduate or professional degree" (i.e., increasing values suggest higher level of education). Blank and "not sure" responses will be coded as missing.

- *School lunch status*. Parents were asked to select their child's lunch status from three options: free school lunch, reduced school lunch, or neither. We will code those who selected neither as 0 and combine both free and reduced-price lunch statuses to code as 1.

Part 3: Data Analysis

1. **Which units of analysis (respondents, cases, etc.) will be included or excluded in your study? Taking these inclusion/exclusion criteria into account, indicate the (expected) sample size of the data you'll be using for your statistical analyses (to the best of your knowledge).**

The expected sample size for the current project's statistical analyses is based on the initial data collection procedures from the larger grant. The estimated number of grade 1 students that we have collected data from is about 600 students. There are missing data based on student absences and attrition between waves. We expect that these absences and attrition will reduce our student sample by approximately 10-15% with an estimated final analytic sample of 528-540 students.

2. **What do you know about missing data in the data set (e.g., overall missingness rate, information about differential dropout)? How will you deal with incomplete or missing data? Based on this information, provide a new expected sample size.**

Planned missing data collection methods were employed in the assessment of several student-level items, including items from the spatial anxiety scale and the mental rotation task, in order to reduce the time necessary for each student to take the survey (Little & Rhemtulla, 2013). We used a multiform design. To create the multiple forms, we first split up all the items into four blocks containing an equal or close-to-equal number of items for each construct. These four blocks were labeled X, A, B, and C.

All students were presented with block X of items and two other blocks of items from blocks A, B, or C. In total, there were three different forms administered. These three forms were made up of items from X, A, and B (Form 1), X, A, and C (Form 2), or X, B, and C (Form 3). Forms were randomly assigned to each teacher. Thus, each student intentionally was randomly assigned, depending on the teacher whose classroom they were in, to receive around 75% of the survey items.

Due to this planned missing data design, the missing data mechanism is known for the items that were not selected to be presented to students based on the form they received. The missing data pattern for these planned missing data are missing completely at random (MCAR), where missing data are a random subset of the data.

Beyond this planned action in student-level items, little else is known about the overall missing data rate at the item level (i.e., item nonresponse) for students. There will likely be other missing data patterns that appear in the student data, based on short student absences during participation in the study and skipping of survey and test items, that were not intentionally planned. Any multiple responses (MR), no responses (NR), and responses that have unclear intent (UI) are coded as missing for the survey data.

The remaining item nonresponse for student-level missing data may be MCAR, missing at random (MAR; missing due to a known reason also measured in the dataset), or missing not at random (MNAR; missing due to the item itself). First, we will assess whether the missing data for students outside of the planned missing data design are MCAR by conducting Little's MCAR test (Chen & Little, 1999) with the survey items we are using in this study. If these data are not MCAR, they may be MAR or MNAR. However, missing data for these survey items are not expected to be due to to the values on the items themselves (i.e., MNAR). To estimate parameters with the available data with either missing data that are MCAR or MAR, we can use the Full-Information Maximum Likelihood (FIML) estimator in each analysis (Enders, 2001). FIML computes parameter estimates based on the means and variances from the observed portion of the variable (Wothke, 2000).

As for differential dropout of student participants, it is important to note that some students in our sample experienced a weather-related disaster during the time frame of our study. As a result, there are fewer children from the affected areas than from non-affected areas in the Wave 2 data. This has created a larger sample than expected of children who have data in fall 2018 but not spring 2019. We will also test for differential attrition to see if there are systematic differences between students in the sample in the fall and those in the spring.

3. **How will you identify a statistical outlier in your data and what will you do when you encounter them?**

We plan to specifically focus on outliers that are due to error (i.e., data entry error, child response error) in these data. To identify error outliers, we plan to run descriptive statistics on each variable to confirm that there are no impossible values included in the data. If there are data points that are extreme but reflected possible values and we have no reason to believe that they are incorrect (e.g., the mean for the item may be a rating of 2 on a 4-point scale but there were scores of 4 from some individuals), they will be kept in the data set. Any data entry errors for individual data points that we believe are true inaccuracies will be corrected based on looking at the raw data or coded as missing in our final data set and the reasons for data exclusion will been noted.

4. **Provide one or more specific and testable hypotheses for each research question. For each hypothesis, describe the statistical model you will use to test the hypothesis.**

Research Question 1: What is the nature of the relation between spatial and math skills across two waves of data collection? Specifically, do we find evidence of early spatial skills predicting later math skills, early math skills predicting later spatial skills, or both (indicating bidirectional relations)? Do spatial and math skills have a unique relation outside of reading skills?

- **Aim 1**: To examine the relation between spatial and math skills over two waves of data collection and, more specifically, examine if early spatial skills predict later

math skills, early math skills predict later spatial skills, or both (indicating bidirectional relations).

  - **Hypothesis for Aim 1**: Based on existing literature, we would expect to see positive statistically significant relations between spatial and math skills within and across waves of data collection (Geer et al., 2019; Lombardi et al., 2019; Verdine et al., 2017). There is some debate as to the causal direction of the relation between spatial and math skills, with some work only finding early spatial skills predicting later math skills (Verdine et al., 2017) and some work finding evidence of bidirectional relations (Geer et al., 2019; Lombardi et al., 2019). Based on this mixed evidence, we expect to find that either early spatial skills predict later math skills, early math skills predict later spatial skills, or that both of these are true.
  - **Data Analysis for Aim 1**: We will conduct a cross-lag panel model with spatial and math skills at Wave 1 predicting spatial and math skills at Wave 2. In doing so, we will be able to address how these constructs are related concurrently at each time point, while also accounting for how they may be related over time with particular interest in the direction of this causal link. We will include gender and SES as covariates.

- **Aim 2**: To address if the relations between spatial and math skills remain significant when accounting for reading skills.
  - **Hypothesis for Aim 2**: Some research has suggested that the relation between spatial and math skills may rely on the shared cognitive resources it takes to complete these tasks (Atit et al., under review; Hawes et al., 2019; Mix et al., 2016). As such, we wanted to include another cognitive skill measure in the model to see if, perhaps, its inclusion would change the significance of the relations between spatial and math skills. Based on findings from Atit et al. (under review), we expect there to be a unique relation between spatial and math skills over and above other cognitive skills (in this case: reading skills)
  - **Data Analysis for Aim 2**: We will conduct the same cross-lag panel model from Aim 1 but will include the measure reading skills (the TOSREC) in the model at Wave 1 as a predictor of Wave 2 math and spatial skills. The goal in including this measure is to examine if adding early reading as a predictor of math and spatial skills at Wave 2 changes the results of the original model. Specifically, we will see if any of the paths between spatial and math skills that were significant in Aim 1 become non-significant once reading in included. We will include gender and SES as covariates.

Research Question 2: Do the relations between spatial and math anxiety at Wave 1 and spatial and math skills at Wave 2 exhibit domain specificity? Specifically, is spatial anxiety at Wave 1 more predictive of spatial skills at Wave 2 than it is of math skills at Wave 2 and is the reverse true for math anxiety? As a secondary aim, do any relations between domain-specific anxieties and cognitive skills remain significant when reading skills are accounted for?

- **Aim 1**: to identify if spatial and math anxiety at Wave 1 predict spatial and math skills at Wave 2, with particular interest in examining if cognitive anxieties exhibit domain-specificity (i.e., math anxiety being more predictive of math skills than spatial skills) in their relations with children's cognitive performance.
    - **Hypothesis for Aim 1**: In a recent study, Lauer et al. (2018) examined the relation between domain-specific anxieties (math, spatial, and verbal anxieties) and their respective cognitive skills in young children. Zero-order correlations between these constructs demonstrated a strong link between spatial and math skills, with some evidence for domain specificity in that spatial anxiety was significantly related to spatial skills and not significantly related to math skills. Math anxiety was significantly related to both math and spatial skills; however, the relation between math anxiety and spatial skills was weaker than that for math anxiety and math skills. In regression analyses, they found that both math and verbal anxieties were significant predictors of math performance, but spatial anxiety was not. Importantly, they found that none of the individual anxieties uniquely predicted spatial reasoning. As such, the results from this study suggest partial support for domain specificity, but that the pattern may look different when the multiple anxieties are included together (Lauer et al., 2018). We think that the relations between cognitive anxieties and cognitive skills may be domain-specific at young ages; however, the Lauer et al. paper is currently the only one to examine this possibility and the evidence is somewhat mixed. Specifically, we have two hypotheses: (1) we expect to find that there will be a stronger relation between math anxiety and math skills than between math anxiety and spatial skills and (2) we expect to find that there will be a stronger relation between spatial anxiety and spatial skills than between spatial anxiety and math skills.
    - **Data Analysis for Aim 1**: We will conduct a cross-lag panel model with spatial and math skills at Wave 1 predicting spatial and math skills at Wave 2. This model will also include spatial and math anxiety at Wave 1 as predictors of Wave 2 spatial and math skills. As with the other models, this model will include gender and SES as covariates. To assess if the domain-specific relations between each cognitive anxiety and its relative skill are stronger than cross-domain relations between cognitive anxieties and skills, we will conduct a series of Wald test models. Specifically, we will run different versions of the main model constraining different paths to be equivalent and see if the Wald test is significant. If constraining the domain-specific paths (i.e., spatial anxiety → spatial skills; math anxiety → math skills) to be equivalent to their relative cross-domain paths (spatial anxiety → math skills and math anxiety → spatial skills, respectively) leads to a significantly worse model fit, then we know that there is a significant difference between the strength of domain-specific and cross-domain relations, the magnitude of which would determine if we saw stronger links between domain-specific anxieties and their relevant cognitive skill.

- **Aim 2**: to address if any relations between cognitive anxieties and skills remain significant when accounting for reading skills.
    - **Hypothesis for Aim 2**: As mentioned above in Research Question 1, Aim 2, some research has suggested that the relation between spatial and math skills may rely on the shared cognitive resources it takes to complete these tasks (Atit et al., under review; Hawes et al., 2019; Mix et al., 2016). As such, we wanted to include another cognitive skill measure in the model to see if, perhaps, its inclusion would change the strength of the relation between spatial and math skills. There is no known literature examining the impact of reading skills on the relations between domain-specific anxieties; however, we would expect that any significant relations would remain significant with reading accounted for.
    - **Data Analysis for Aim 2**: We will conduct the same cross-lag panel model from Research Question 2 Aim 1 but will include the measure of reading skills (the TOSREC) in the model at Wave 1 as a predictor of Wave 2 math and spatial skills. The goal in including this measure is to examine if adding early reading as a predictor of math and spatial skills at Wave 2 changes the results of the original model. Specifically, we will test if any of the initially significant paths between spatial and math anxiety and spatial and math skills become non-significant once reading in included. We will include gender and SES as covariates.

5. **What criteria will you use to make inferences? Will you be using one- or two-tailed tests for each of your analyses? If you are comparing multiple conditions or testing multiple hypotheses, will you account for this, and if so, how?**

We will report specific model fit indices of chi-square (χ^2), root mean square error of approximation (RMSEA), comparative fit index (CFI), and Tucker-Lewis index (TLI) for each model. To assess the magnitude of the relation between variables (within and across time points) we will use *p*-values and standardized regression coefficients. The *p*-values are two-tailed, and we will consider *p*-values below .05 to indicate statistical significance, when relevant.

For Research Question 2, to assess if the domain-specific paths (e.g., Wave 1 spatial anxiety → Wave 2 spatial skills) are significantly stronger than the cross-domain paths (e.g., Wave 1 spatial anxiety → Wave 2 math skills), we will conduct Wald Tests. The results of the Wald Test will be a chi-square (χ^2) distributed test statistic and *p*-value, which will be interpreted in the final paper for each Wald Test conducted.

6. **What will you do should your data violate assumptions, your model not converge, or some other analytic problem arises?**

To account for the fact that our data violate the assumption of independence due to students being taught in the same classroom being more similar to one another, we used maximum likelihood estimation with robust standard errors with the type = complex command in MPlus. We will accommodate any other unforeseen analytic problems as best we can when they arise and use the current best research practices for addressing them.

7. **Provide a series of decisions about evaluating the strength, reliability, or robustness of your focal hypothesis test.**

If we feel the assumptions of CLPM are not met, we will consider running a latent change score model to see if the results are consistent. In addition, if there are issues with multicollinearity between predictor variables, we will also assess predictive models with predictors or covariates removed.

8. **If you plan to explore your data set to look for unexpected differences or relationships, you can describe those tests here, or add them to the final paper under a heading that clearly differentiates this exploratory part of your study from the confirmatory part.**

If any analyses are conducted beyond the ones discussed in this preregistration, they will be indicated as exploratory analyses in the manuscript. For example, we may wish to explore if there are any gender differences in the relations between cognitive skills and anxieties, due to the focus in existing literature on gender differences in spatial cognition and math performance. This analysis would involve comparing the relations between spatial and math skills for boys versus girls, to see if the model results are similar across genders. Specifically, this would involve conducting model comparison analyses that allow us to compare the model results in two independent samples (boys vs. girls).

Part 4: Data Access and Knowledge

1. **Specify whether this data is open or publicly available. If not publicly accessible, how will materials/data be available for reproducibility by other researchers?**

Data for the larger grant-funded project are not yet open or publicly available. These data will be made available by Summer 2023 (approximately 1 year after the end of the grant period) as specified in the Data Management Plan for the funding agency.

2. **Specify the date of download and/or access of the data.**

Data will be accessed in May 2021 after this preregistration has been submitted.

3. **What prior knowledge do you have at the time of preregistration about trends in the data set you will be working with? Are there any publications, conference presentations (papers, posters), and working papers (in prep, unpublished, preprints) that are based on the data set.**

The data specifically being used for the project in this preregistration were analyzed by the first author for a conference presentation; however, the math achievement and spatial skills data were not in their final form and the analyses conducted for that presentation do not match onto the analyses planned for the current project.

The second author of this preregistration has conducted preliminary analyses (unrelated to the present study's goals or analyses) for a conference, however, the data used for the present study were not included. Additionally, the math achievement data were not in their final form of a theta score that will be used for this study. Other researchers on the research team are using other parts of the dataset that do not overlap with the variables used here other than the demographic information.

References

Atit, K., Power, J. R., Pigott, T., Lee, J., Geer, E. A., Uttal, D., Ganley, C. M., & Sorby, S. (under review). Examining the Relations Between Spatial Skills and Mathematics Performance: A Meta-Analysis.

Chen, H. Y., & Little, R. (1999). A test of missing completely at random for generalised estimating equations with missing data. *Biometrika*, *86*(1), 1-13.

Enders, C. K. (2001). The impact of nonnormality on full information maximum-likelihood estimation for structural equation models with missing data. *Psychological methods*, *6*(4), 352.

Ganley, C. M., & McGraw, A. L. (2016). The development and validation of a revised version of the math anxiety scale for young children. *Frontiers in Psychology*, *7*, 1181. doi: 10.3389/fpsyg.2016.01181

Geer, E. A., Quinn, J. M., & Ganley, C. M. (2019). Relations between spatial skills and math performance in elementary school children: A longitudinal investigation. *Developmental Psychology*, *55*(3), 637.

Harari, R. R., Vukovic, R. K., & Bailey, S. P. (2013). Mathematics anxiety in young children: an exploratory study. *The Journal of Experimental Education*, *81*(4), 538-555. doi: doi.org/10.1080/00220973.2012.727888

Hawes, Z., Moss, J., Caswell, B., Seo, J., & Ansari, D. (2019). Relations between numerical, spatial, and executive function skills and mathematics achievement: A latent-variable approach. *Cognitive Psychology*, *109*, 68-90. https://doi.org/10.1016/j.cogpsych.2018.12.002

Lauer, J. E., Esposito, A. G., & Bauer, P. J. (2018). Domain-specific anxiety relates to children's math and spatial performance. *Developmental psychology*, *54*(11), 2126.

Little, T. D., & Rhemtulla, M. (2013). Planned missing data designs for developmental researchers. *Child Development Perspectives*, *7*(4), 199-204.

Lombardi, C. M., Casey, B. M., Pezaris, E., Shadmehr, M., & Jong, M. (2019). Longitudinal analysis of associations between 3-D mental rotation and mathematics reasoning skills during middle school: Across and within genders. *Journal of Cognition and Development*, *20*(4), 487-509.

Mix, K. S., Levine, S. C., Cheng, Y., Young, C., Hambrick, D. Z., Ping, R., & Konstantopoulos, S. (2016). Separate but correlated: The latent structure of space and mathematics across development. *Journal of Experimental Psychology: General, 145*(9), 1206-1227. doi:10.1037/xge0000182

Ramirez, G., Gunderson, E. A., Levine, S. C., & Beilock, S. L. (2012). Spatial anxiety relates to spatial abilities as a function of working memory in children. *The Quarterly Journal of Experimental Psychology*, *65*(3), 474-487.

Schoen, R. C., Anderson, D., Riddell, C. M., & Bauduin, C. (2018a). Elementary Mathematics Student Assessment: Measuring the performance of grade 3, 4, and 5 students in number (whole numbers and fractions), operations, and algebraic thinking in fall 2015 (Research Report No. 2018-24). Tallahassee, FL: Florida State University.

Schoen, R. C., Anderson, D., & Bauduin, C. (2018b). Elementary Mathematics Student Assessment: Measuring grade 3, 4, and 5 Students' Performance in Number (Whole Numbers and Fractions), Operations, and Algebraic Thinking in Spring 2016. (Research Report No. 2018-23). Tallahassee, FL: Florida State University.

Thurstone, L. L. (1974). A law of comparative judgment. *Scaling: A sourcebook for behavioral scientists*, 81-92.

Treiman, D. J. (1976). A standard occupational prestige scale for use with historical data. *The Journal of Interdisciplinary History*, *7*(2), 283-304.

Verdine, B. N., Golinkoff, R. M., Hirsh-Pasek, K., & Newcombe, N. (2017). *Links between spatial and mathematical skills across the preschool years*. Hoboken: Wiley

Wagner, R. K., Torgesen, J. K., Rashotte, C. A., Pearson, N. A. (2010). Test of Silent Reading Efficiency and Comprehension (TOSREC) examiner's manual. Austin, TX: Pro-Ed.

Wothke, W. (2000). *Longitudinal and multigroup modeling with missing data.* In T. D. Little, K. U. Schnabel, & J. Baumert (Eds.), *Modeling longitudinal and multilevel data: Practical issues, applied approaches, and specific examples* (pp. 219–240, 269–281). Lawrence Erlbaum Associates Publishers.
