# Supplementary material for: Bidirectional Relations Between Spatial and Mathematics Skills in Elementary School Children: The Role of Domain-Specific Anxieties
Source: J Intell. 2026 May 19;14(5):87. doi: 10.3390/jintelligence14050087 (PMC13207808; doi:10.3390/jintelligence14050087)
Supplement: Supplementary file 1 [file jintelligence-14-00087-s001.zip › Supplementary Figures.pdf]

Supplemental Materials

Robustness Check: Including Reading as a Covariate

Correlational Results

There were significant moderate to strong correlations between reading skills and math skills at both Wave 1 ($r = .49$, $p < .001$) and Wave 2 ($r = .37$, $p < .001$) as well as significant weak correlations with spatial skills at Wave 1 ($r = .16$, $p < .001$) and Wave 2 ($r = .18$, $p < .001$). Interestingly, there was also a significant small negative correlation between reading skills and math anxiety ($r = -.14$, $p < .001$), but there was no significant correlation between reading skills and spatial anxiety ($r = -.01$, $p = .882$).

Research Question 1

As a secondary aim of Research Question 1, we conducted a robustness check on our initial model to examine if any relations between spatial and math skills remained significant when accounting for reading skills at Wave 1. To test this, we conducted the same cross-lagged panel model from Research Question 1 but included a measure of reading skills at Wave 1 as a predictor (see Figure S1). The pattern of results from the previous model remained the same, still showing significant bidirectional relations between math and spatial skills. In addition, we found that reading skills were significantly correlated with both math skills at Wave 1 ($r = .39$, $p < .001$) and spatial skills at Wave 1 ($r = .17$, $p = .001$). These results suggest that accounting for reading skills does not account for the relations between spatial and math skills over two waves of data collection.

Research Question 2

As a secondary aim of Research Question 2, we conducted a robustness check on our initial model to examine if any relations between math and spatial anxieties and

skills remain significant when accounting for reading skills at Wave 1. To do this, the same cross-lagged panel model from Research Question 2 was run with a measure of reading skills added at Wave 1 (Figure S2). The pattern of results was the same as the first version of this model, with additional statistically significant correlations that emerged between reading skills and math skills at Wave 1 ($r = .50, p < .001$), reading and spatial skills ($r = .17, p = .001$), as well as reading skills and math anxiety ($r = -.14, p = .008$). Reading was not significantly correlated with spatial anxiety ($r = -.01, p = .766$). Once again, two Wald Tests were conducted to examine if the domain-specific relation for either cognitive anxiety is significantly stronger than the cross-domain relation when accounting for reading skills. The first Wald Test examined if the path between math anxiety and Wave 2 math skills was significantly stronger than the path between math anxiety and Wave 2 spatial skills. Results showed that the domain-specific path was significantly stronger than the cross-domain path ($\Delta\chi^2 = 7.87$, $df = 1$, $p = .005$), suggesting domain specificity for math anxiety as it relates to later skills. The second Wald Test examined if the reverse was true for spatial anxiety and results demonstrated that there was no significant difference between the domain specific and cross-domain pathways for spatial anxiety ($\Delta\chi^2 = .13$, $df = 1$, $p = .718$), suggesting that spatial anxiety is not exhibiting domain specificity at this age. These results support the contention that adding reading skills into the model as a predictor does not change the relations between math and spatial anxiety and their respective skills.

Exploratory Sensitivity Analysis: Latent Modeling and Error Correction

We also conducted analyses with mental rotation skills modeled as a latent construct, and additional analyses that also had measurement error correction for the mathematics skills measure as well. Results of these analyses are presented below in Figures S3-S6 and summarized in the main manuscript.

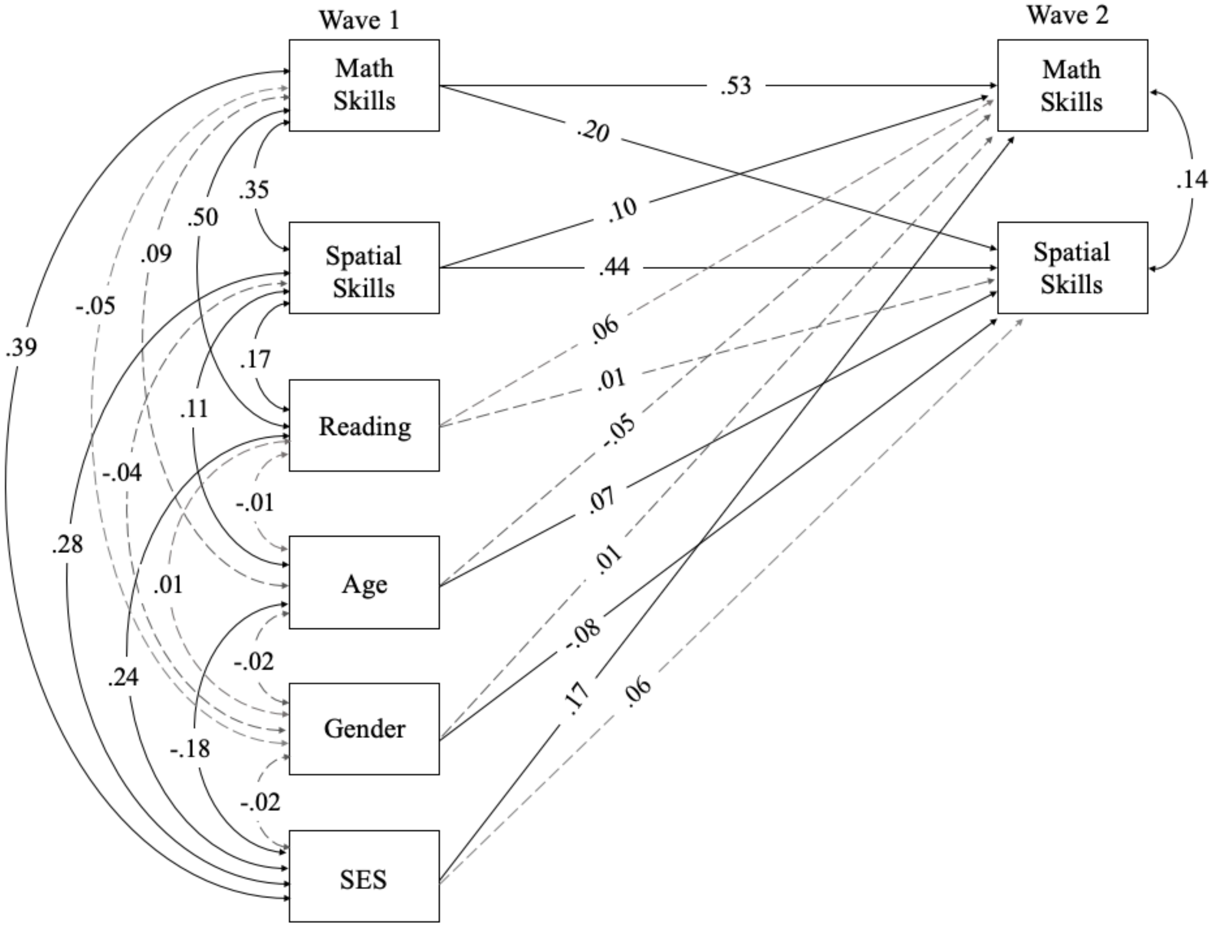

Figure S1. Model for Research Question 1 Aim 2: Relation between Spatial and Math Skills with Reading. For this model, black solid lines denote significant results ($p < .05$) and grey dashed lines denote non-significant results ($p > .05$). SES refers to socioeconomic status.

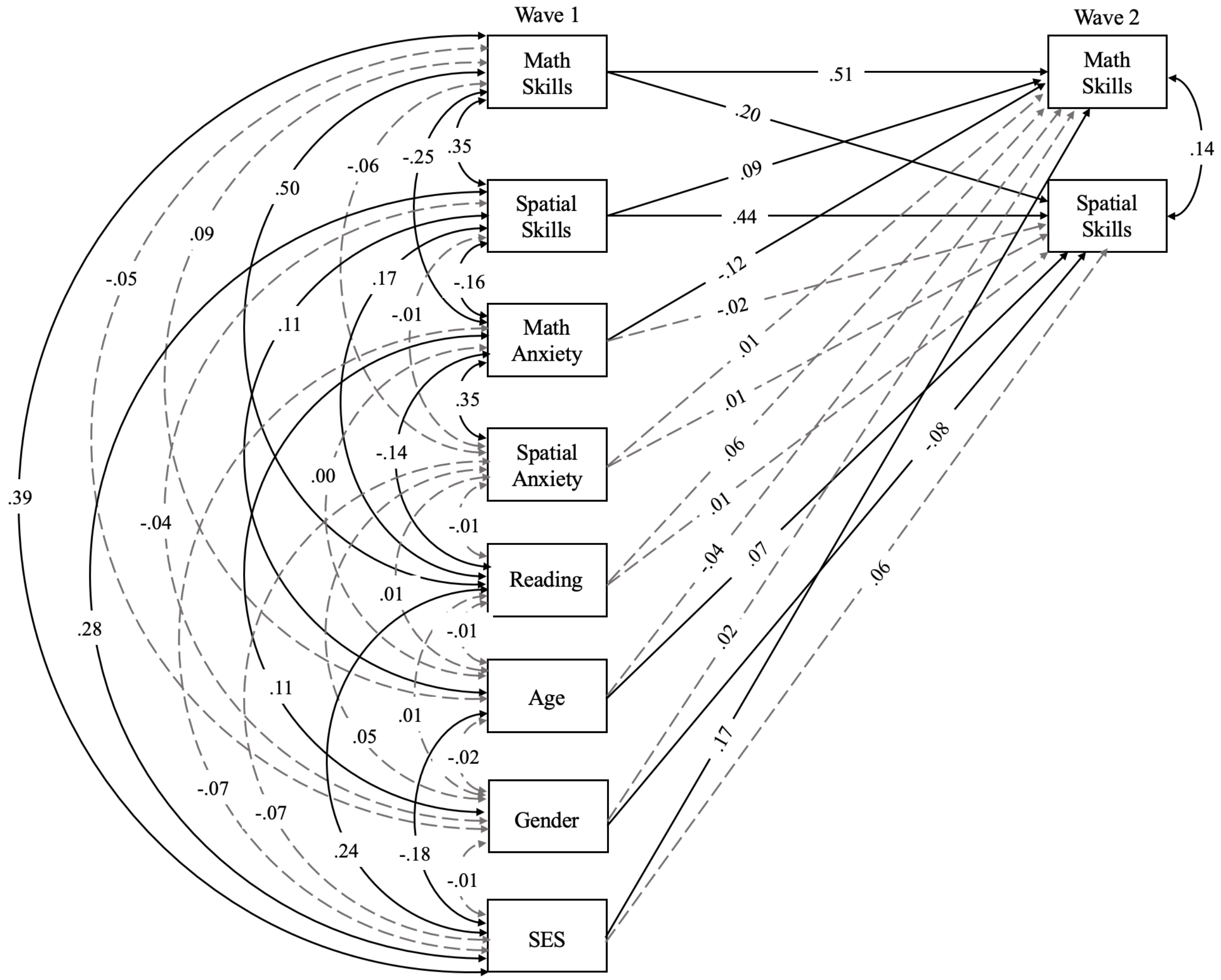

Figure S2. Model for Research Question 2 Aim 2: Relation between Cognitive Anxieties and Skills with Reading. For this model, black solid lines denote significant results ($p < .05$) and grey dashed lines denote non-significant results ($p > .05$). SES refers to socioeconomic status.

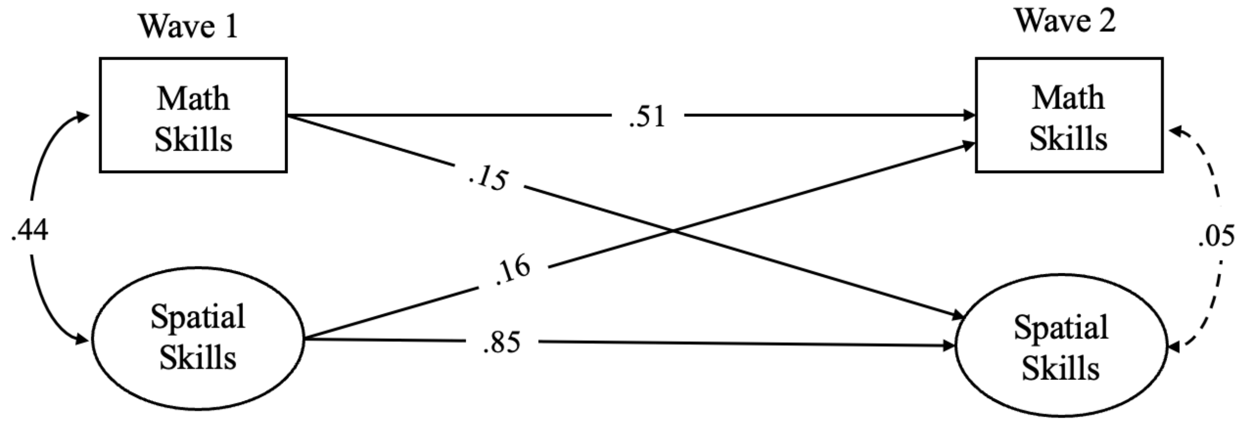

Figure S3. Model for Exploratory Sensitivity Analysis for Research Question 1 Aim 1 with spatial skills modelled as latent factors. For this model, solid lines denote significant results ($p < .05$) and dashed lines denote non-significant results ($p > .05$). Please note, this is a simplified version of this model that reflects changes only to key findings.

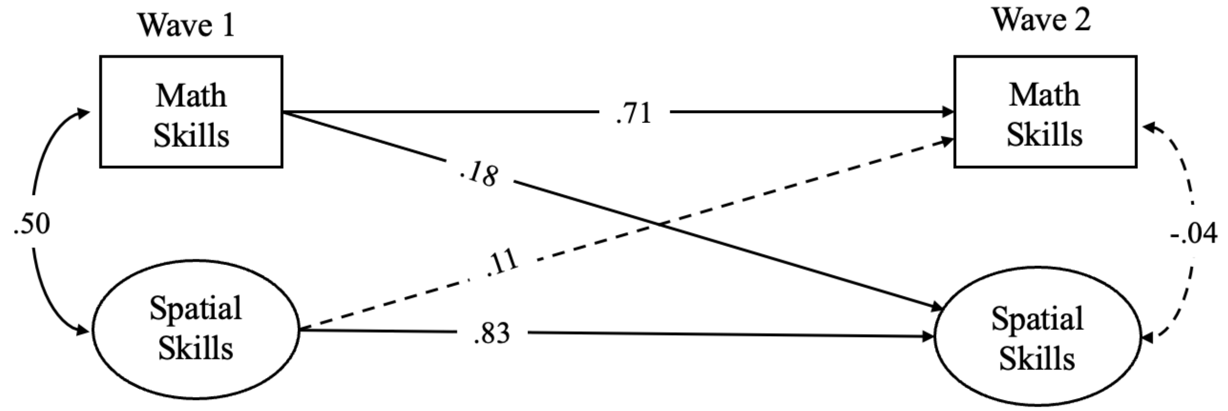

Figure S4. Model for Exploratory Sensitivity Analysis for Research Question 1 Aim 1 with spatial skills modelled as latent factors and measurement error corrections implemented for mathematics skills. For this model, solid lines denote significant results ($p < .05$) and dashed lines denote non-significant results ($p > .05$). Please note, this is a simplified version of this model that reflects changes only to key findings.

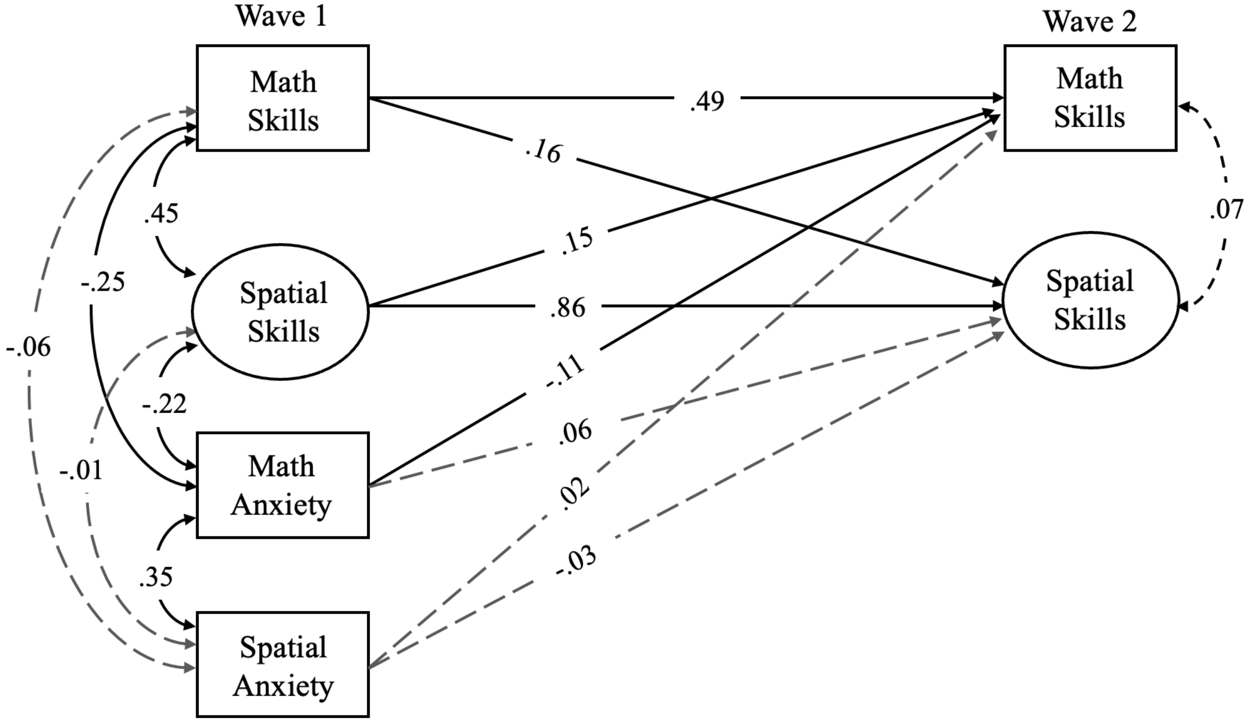

Figure S5. Model for Exploratory Sensitivity Analysis for Research Question 2 Aim 1 with spatial skills modelled as latent factors. For this model, solid lines denote significant results ($p < .05$) and dashed lines denote non-significant results ($p > .05$). Please note, this is a simplified version of this model that reflects changes only to key findings.

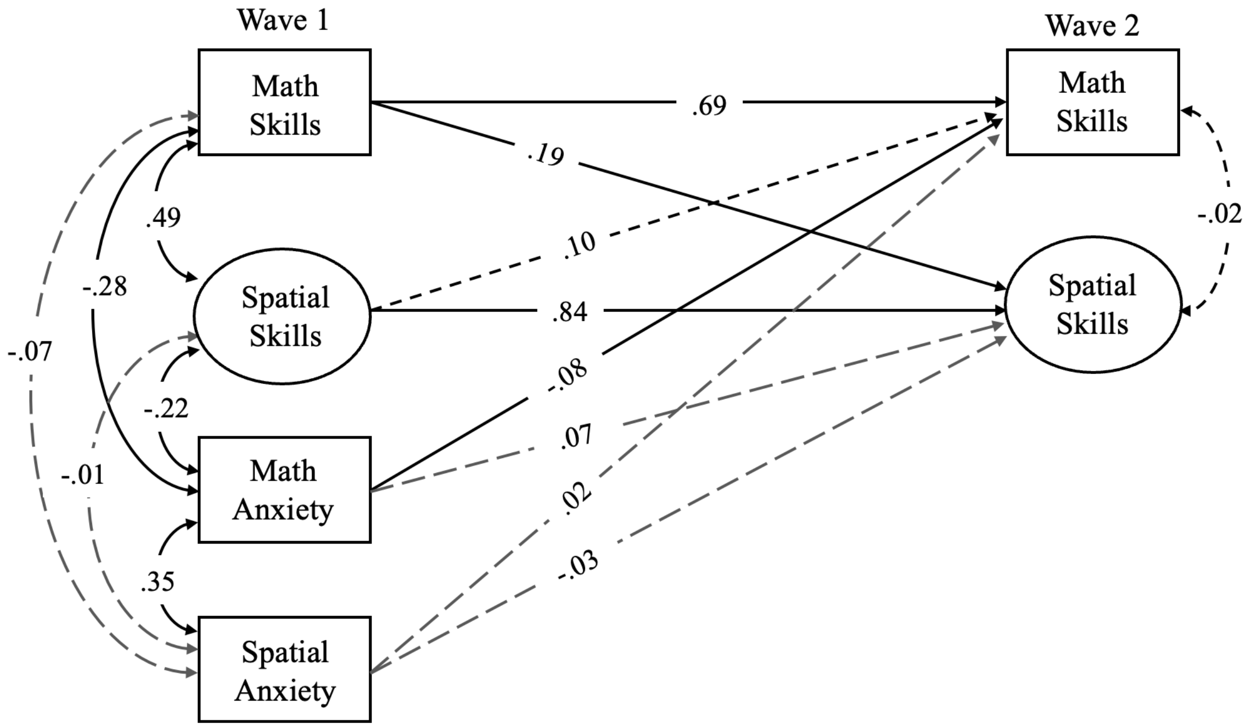

Figure S6. Model for Exploratory Sensitivity Analysis for Research Question 2 Aim 1 with spatial skills modelled as latent factors and measurement error corrections implemented for mathematics skills. For this model, solid lines denote significant results ($p < .05$) and dashed lines denote non-significant results ($p > .05$). Please note, this is a simplified version of this model that reflects changes only to key findings.
